# Duck PIAS2 Promotes H5N1 Avian Influenza Virus Replication Through Its SUMO E3 Ligase Activity

**DOI:** 10.3389/fmicb.2020.01246

**Published:** 2020-06-11

**Authors:** Shaopo Zu, Qian Xue, Zhuoliang He, Chenxi Shi, Junsheng Zhang, Wenbo Wu, Weiqiang Li, Zhiting Liu, Jianni Huang, Peirong Jiao, Ming Liao

**Affiliations:** ^1^College of Veterinary Medicine, South China Agricultural University, Guangzhou, China; ^2^College of Veterinary Medicine, Northeast Agricultural University, Harbin, China

**Keywords:** H5N1 avian influenza virus, duck, protein inhibitor of activated STAT2, nucleoprotein, protein–protein interactions

## Abstract

The protein inhibitor of the activated STAT2 (PIAS2) has been implicated in many cellular processes and can also regulate viral replication in mammals. However, the role of PIAS2 in the highly pathogenic avian influenza virus (HPAIV) H5N1 replication in ducks is still unclear. Through liquid chromatography–tandem mass spectrometry (LC-MS/MS) assay, we identified that duck PIAS2 (duPIAS2) was one protein that interacted with the nucleoprotein (NP) from the H5N1 HPAIV strain of DK212. Through confocal microscopy images and Co-IP assay, we confirmed NP could interact with duPIAS2. Overexpression of duPIAS2 in primary duck embryo fibroblast (DEF) cells was shown to promote DK212 replication, and knockdown of duPIAS2 could repress DK212 replication. We further found duPIAS2 could promote NP SUMOylation through duck SUMO1 (duSUMO1), and the potential SUMOylation sites of NP were at lysines 7, 48, and 87. Furthermore, duPIAS2 promoted the replication of DK212, here relying on the activity of its SUMO E3 ligase. Duck SENP1 (duSENP1), a deSUMOylation enzyme, could repress NP SUMOylation and also inhibit DK212 replication. Together, we identified duPIAS2 could interact with NP and that duPIAS2 promoted H5N1 HPAIV replication, which might be related to NP SUMOylation.

## Introduction

Avian influenza virus (AIV), a member of the influenza A virus, is classified as highly pathogenic avian influenza virus (HPAIV) or low pathogenic avian influenza virus (LPAIV) based on its pathogenicity in chickens (Horimoto and Kawaoka, 2005). H5N1 HPAIV often causes a high mortality rate in birds and occasionally infects humans. To date, 860 laboratory-confirmed human H5N1 infections have been reported, including 454 deaths (2019, WHO). Indeed, AIVs have caused devastating losses to the poultry industry, and they still threaten public health (Li et al., 2004).

The influenza A virus is a negative-sense RNA virus that belongs to the Orthomyxoviridae family. Its genome consists of eight segments of single-stranded viral RNA (vRNA). Each vRNA segment is encapsulated by multiple nucleoproteins (NPs) and a trimeric RNA polymerase complex (PB1, PB2, and PA) that form the viral ribonucleoprotein (vRNP) (Lamb and Choppin, 1983). Among influenza A viruses, NP is a conserved structural protein and plays multiple essential functions during viral infection, including vRNA replication, transcription, vRNP transportation, and viral genome packing (Ye et al., 2007). To complete these functions, NP must interact with lots of host factors and utilize the host post-translational modification (PTM) machinery to regulate its functions in different phases of viral infection (Watanabe et al., 2014; Eisfeld et al., 2015; Giese et al., 2017). WSN H1N1 NP is a monoubiquitinated protein and can be deubiquitinated by deubiquitinating enzyme USP11 through interacting with USP11, which is crucial for viral genome replication (Liao et al., 2010). NP could be acetylated at several lysine residues, and constant acetylation or non-acetylation at the K229 site of NP represses viral release (Giese et al., 2017). During viral replication, NP shuttles between the cytoplasm and nucleus, which could be controlled by phosphorylation and dephosphorylation of NP (Zheng et al., 2015).

Like ubiquitination, SUMOylation is also a reversible covalent PTM in which the lysine residues of target proteins are attached by small ubiquitin-related modifiers (SUMOs) through specialized enzymatic cascades, which require the E1 activating enzyme, E2 conjugating enzyme, and E3 ligase enzyme (Kerscher et al., 2006; Capili and Lima, 2007). It has been reported that SUMO1, SUMO2, and SUMO3 are ubiquitously expressed in humans (Eaton and Sealy, 2003). SUMO1 shares a 45% sequence identity to SUMO2/3, but SUMO2 and SUMO3 share a 97% sequence identity (Geiss-Friedlander and Melchior, 2007). SUMO1 and SUMO2/3 are conjugated to different target proteins and serve different functions (Tatham et al., 2001). SUMOylated proteins participate in multiple processes both in the cytoplasm and nucleus, including gene transcriptional regulation, DNA repair, RNA processing, and cell cycle progression (Johnson, 2004; Geiss-Friedlander and Melchior, 2007; Gareau and Lima, 2010). It has been reported that viral infection could trigger the promotion or reduction of host SUMOylation (Wilson and Rangasamy, 2001). Influenza virus infection causes host SUMO redistribution, which is involved in numerous cellular processes (Domingues et al., 2015). The SUMOylation process could also be manipulated by a virus to promote the virus replication during infection (Everett et al., 2013). Some encoding proteins of the influenza virus are SUMOylated during viral infection, which is beneficial to viral trafficking, assembly, and morphogenesis (Wu et al., 2011; Han et al., 2014). The Ebola VP24 protein is modified by SUMO, which contributes to the repression of type I interferon expression and blocking of IFN-mediated STAT1-nuclear translocation (Vidal et al., 2019).

The protein inhibitor of activated STAT (PIAS), a SUMO E3 ligase, is initially found as a repressor of the STAT pathway before participating in multiple cellular processes, including promyelocytic leukemia protein (PML) stability, DNA repair, oncogenesis, immune regulation, and cell proliferation (Ivashkiv, 2000; Shuai and Liu, 2005). The RING-finger-like zinc-binding domain (RLD) confers PIAS to SUMO E3 ligase activity, and PIAS regulates some cellular processes that are needed by SUMOylation E3 ligase activity in mammal cells (Shuai and Liu, 2005; Kubota et al., 2011; Li et al., 2013). For example, PIAS2 can specifically interact with melanoma differentiation-associated gene 5 (MDA5) and enhance the MDA5 SUMOylation to inhibit vesicular stomatitis virus replication by elevating type I IFN induction in mammal cells (Fu et al., 2011). PIAS2 could SUMOylate Smad4 to stabilize Smad4 to upregulate the transforming growth factor-β-mediated pathway (Ohshima and Shimotohno, 2003). PIAS2 can also regulate viral replication by interacting with viral proteins directly and promoting the SUMOylation of viral proteins. For example, PIAS2 interacts with the papillomavirus helicase E1 protein to stimulate E1 SUMOylation, which influences papillomavirus replication (Rosas-Acosta et al., 2005); PIAS2 interacts with Rta of Epstein-Barr virus (EBV) and then enhances Rta SUMOylation which is important in the EBV lytic cycle (Liu et al., 2006). As expected, how PIAS2 regulates viral replication mainly depends on where the SUMOylated substrates end up. PIAS2 SUMOylated core protein of Hepatitis C virus (HCV) can degrade core proteins, thereby inhibiting HCV replication (Guo et al., 2017). PIAS2α interacts with NP of the WSN H1N1 to promote NP SUMOylation, which ensures the normal trafficking of NP in mammal cells (Han et al., 2014).

PIAS proteins in response to many pathogen infections in mammals and other species have been well-studied (Huang et al., 2015; Brown et al., 2016; Niu et al., 2018). Ducks perpetuate most strains of influenza viruses in nature, often appear asymptomatic when infected with some forms of HPAIV (Cheng et al., 2015a). In addition, birds have a smaller repertoire of immune genes than mammals, and some different events may happen between duck and mammalian cells upon virus infection (Cheng et al., 2019). The relationships between PIAS and H5N1 AIV infection in duck cells arouse our interest. In this study, we found that duPIAS2, a SUMO E3 ligase, was a potential interaction protein for DK212 NP by liquid chromatography–tandem mass spectrometry (LC-MS/MS) screening, and it could promote DK212 replication. duPIAS2 could also interact with NP and promote NP SUMOylation by SUMO1, and lysines 7, 48, and 87 of NP were the potential sites to be SUMOylated. In addition, the SUMO E3 ligase activity of duPIAS2 was necessary for its function in the promotion of DK212 replication.

## Materials and Methods

### Viruses and Cells

A/Duck/Guangdong/212/2004 (H5N1) virus (DK212) was isolated from ducks in Guangdong, China, in 2004 (Wei et al., 2014). The viruses were purified and then propagated in specific pathogen-free (SPF) chicken embryos. Viral titers in 50% tissue culture infectious dose (TCID_50_) were calculated using Reed and Muench’s method. All experiments involving H5N1 HPAIV were conducted in an animal biosafety level 3 (ABSL-3) laboratory.

The human embryonic kidney 293T (HEK 293T) cells and primary duck embryo fibroblast (DEF) cells derived from 10-day-old duck embryos were maintained in Dulbecco’s modified Eagle medium (GIBCO, United States), supplemented with 10% fetal bovine serum (GIBCO, United States), penicillin (100 U/mL), and streptomycin (100 U/mL). The cells were incubated at 37°C, 5% (v/v) CO_2_.

### Plasmids

The coding sequence of duck SUMO1 (XM_027461471.1), SUMO2 (XM_027471718.1), SENP1 (XM_005028698.3), UBC9 (XM_005018620.3), and PIAS (PIAS1: XM_005022957.3; PIAS2: XM_005022330.3; PIAS4: XM_005018676.3) families with Myc, V5, and HA tags were cloned into the pCAGGS vector to obtain duSUMO1-Myc, duSUMO2-Myc, duUBC9-HA, duSENP1-V5, and duPIAS-HA expression plasmids. duSUMO1-Myc and duSUMO2-Myc were constructed with Myc at the N-terminus. duUBC9-HA, duSENP1-V5 and duPIAS2-HA were constructed with tags at the C-terminus. At the end of the 3′ terminus of the duSUMO genes, GG-to-AA mutations were introduced by overlap extension PCR named duSUMO1mu-Myc or duSUMO2mu-Myc, and a W374A mutation was introduced into duPIAS2-HA called duPIAS2mu-HA. The NP coding sequence of DK212 with Flag tag was cloned into pCAGGS and called DK212-NP-Flag. K-to-R mutations were introduced into the NP-encoding genes by using overlap extension PCR with primers containing K-to-R mutations. All NP mutation genes were cloned into pCAGGS to construct NP mutation expression plasmids. All recombinant plasmids were sent for DNA sequencing. The primers are shown in Table 1.

**TABLE 1:** Sequence of primers used in this study.

Primer	Sequence (5’–3’)
duPIAS2-F1	CCAACTACCACTGTGATACC
duPIAS2-R1	GAACCTCTGAATACTGCTCT
duPIAS2-F2	CAACCAGTCACCACGAGAGC
duPIAS2-R2	TTCCCCAGAACAGGCCAGAA
duPIAS2-F3	ATGGTATCAAGTTTTCGTGTGTCT
duPIAS2-R3	TCAGTCCAGTGAGATGATGTCAGG
duPIAS2-C-HA-F1	CGGAATTCATGGTATCAAGTTTTCGTGT
duPIAS2-C-HA-R1	CCCTCGAGTCAAGCGTAGTCTGGGACGTCGTATGG GTAGTCCAGTGAGATGATGTCAG
Q-duPIAS2-F	TGAGATTGCCACAACCAGTCTGC
Q-duPIAS2-R	GACAGGACAGATCCAGGTAGGCTT
duSUMO2-F	GGGGTACCATGGAGCAGAAACTCATCTCTGAAGAG GATCTGGCCGACGAGAAGCCCAAGG
duSUMO2-R	CCCTCGAGTTAGTAAACTCCTCCTGTTTGC
duSUMO2mu-R	CCCTCGAGTTAGTAAACTGCTGCTGTTTGCT
duSUMO1-F	TCCCCCGGGATGGAGCAGAAACTCATCTCTGAAGA GGATCTGTTTTGTTTTCAGGAAGCA
duSUMO1-R	CCCTCGAGCTAAACTGTTGAGTGACCCCCC
duSUMO1mu-R	CCCTCGAGCTAAACTGTTGAGTGAGCCGCCGT
duUBC9-F	TCCCCCGGGATGTCTGGCATAGCTCTCAGT
duUBC9-R	CCCTCGAGTTAAGCGTAGTCTGGGACGTCGTATGG GTATGATGGTGCAAACTTCTTGGCTT
duSENP1-F	TCCCCCGGGATGGGTAAGCCTATCCCTAACCCTCTC CTCGGTCTCGATTCTACGCAGAGCTGTGATCTCTCC
duSENP1-R	CTAGCTAGCTCATAGCAGCTTGCGGTGCA
T-PCAGGS-F	TAACCATGTTCATGCCTTCTT
T-PCAGGS-R	TTTATTAGCCAGAAGTCAGAT
Q-duGAPDH-F	ATGTTCGTGATGGGTGTGAA
Q-duGAPDH-R	CTGTCTTCGTGTGTGGCTGT

### The Expression of duPIAS2 in DEF Cells Infected With DK212

DEF cells were seeded in six-well plates and cultured until 90% confluence. Then, the cells were infected with 10 or 100 TCID_50_/well of DK212. The cells were collected at 12 and 24 hpi (hour post-infection), and RNA was extracted using an RNA extraction kit (Fastagen, China) according to the manufacturer’s instructions. A qRT-PCR assay was carried out to measure the relative expression of duPIAS2 with the primers Q-duPIAS2-F and Q-duPIAS2-R (Table 1). qRT-PCR assay using the SYBR Green I PCR kit (Qiagen, Germany) was performed in the Bio-Rad CFX96 Touch^TM^ Real-Time PCR Detection System. qRT-PCR conduction was done with an initial denaturation at 95°C for 30 s, followed by 40 cycles of denaturation at 95°C for 5 s, and an annealing and extension at 60°C for 30 s. The 2^–ΔΔCt^ method was used to analyze the relative expression of the duPIAS2 gene. Duck GAPDH, a useful reference gene, was used to normalize qRT-PCR in the tested group. The qRT-PCR assay was done in triplicate (three wells) and then the whole experiment was repeated three times.

### Overexpression of duPIAS2 Promotes DK212 Replication

DEF cells were seeded in six-well plates and cultured until 70% confluence. Then, the cells were transfected with 4 μg duPIAS2-HA or an empty vector using Hieff Trans (Yeasen, China) according to the manufacturer’s directions. At 24 h post-transfection, 100 TCID_50_/well DK212 was inoculated to DEF cells and adsorbed for 1 h. Cell supernatant was collected at 12, 24, 36, and 48 hpi, and viral titers were detected by TCID_50_ assay.

### Knockdown of duPIAS2 Inhibits DK212 Replication

Three shRNAs targeting duPIAS2 were designed (Genepharma, China), and the target sequences were as follows: shPIAS2-921: GCAGTGCCAAACCAGATTTCC; shPIAS2-1649: GCCCAGC GTGACAACGGTTGA; and shPIAS2-1945: GCAGTGCTCCT GACATCATCT. DEF cells were seeded in six-well plates and cultured until 70% confluence. Then, the cells were transfected with 4 μg shRNA or shNC (negative control) using Hieff Trans. After 36 h, qRT-PCR assay was used to measure the knockdown efficiency of duPIAS2. To study the effect of duPIAS2 knockdown on the replication of AIV, 100 TCID_50_/well DK212 was used to infect shRNA-treated DEF cells. Supernatants were collected at 12, 24, 36, and 48 hpi, and viral titers were measured by TCID_50_ assay.

### Coimmunoprecipitation and Western Blotting

The coimmunoprecipitation assay was performed in HEK 293T cells systems as a previous study (Cheng et al., 2015b). In brief, HEK 293T cells were cultured until 80% confluence and then transfected with duPIAS2-HA and DK212-NP-Flag using Hieff Trans. At 36 h post-transfection, the cells were washed with cold PBS and lysed with an IP Western lysis buffer containing 20 mM Tris (pH 7.5), 150 mM NaCl, 1% Triton X-100, 1 mM EDTA (Beyotime, China), and 1% PMSF (Yeasen, China) for 20 min on ice. The cell lysate was centrifuged at 14,000 g at 4 °C for 15 min. The supernatant was incubated with 50 μL agarose beads (Thermo Fisher Scientific, United States) and rocked for 6 h at 4°C. The beads were washed with TBST three times after incubation and boiled in 2^∗^SDS Buffer (Dingguo, China) for 10 min. Protein samples were subjected to Western blot. NP-Flag and duPIAS2-HA were detected using anti-NP mouse monoclonal antibodies (Sino Biological, China) and anti-HA rabbit polyclonal antibodies (Sigma, United States), respectively. The secondary antibodies were DyLight 800 goat antimouse and antirabbit IgG (H+L) (Thermo Fisher Scientific, United States). The membranes were imaged using an Odyssey infrared imaging system (Li-CoR, United States).

### Identification of duPIAS2-Interacting Proteins Using Affinity Precipitation and LC-MS/MS

DEF cells were seeded in 10 cm dishes and cultured until 70% confluence. Then, the cells were transfected with 20 μg pCAGGS-212NP-Flag or 20 μg pCAGGS-eGFP-Flag using with Hieff Trans. At 36 h post-transfection, the DEF cells were lysed with an IP Western lysis buffer, and then, the supernatant of the lysate was incubated with anti-Flag agarose beads. Next, immunoprecipitated protein complexes were separated by SDS-PAGE assay, and Coomassie blue (Thermo Fisher Scientific, United States) was used to stain the separation gel. Finally, these protein samples were performed by (LC-MS/MS) analysis (PTM Biolabs, China).

### Confocal Microscopy

To study the colocalization of duPIAS2 and DK212-NP, HEK 293T cells were seeded on coverslips in 24-well plates and cotransfected with duPIAS2-eGFP with green fluorescence and with NP-AsRed with red fluorescence using Hieff Trans. At 36 h post-transfection, the transfected cells were washed with PBS and then fixed with 4% paraformaldehyde for 10 min. The fixed cells were permeabilized with 0.1% Triton X-100 for 10 min at 4°C. After washing three times, the treated cells were stained with 1 μM 4′, 6-diamidino-2-phenylindole (DAPI) for 5 min. Fluorescent images were acquired using a TCS SP8 confocal microscope (Lecia, Germany) under a ×100 oil objective.

### Statistical Analysis

Data are presented as means ± SD, and GraphPad Prism 7 software (GraphPad Software Inc., United States) was used to perform the statistical analyses. A student’s *t*-test was used to evaluate the differences among the groups, two-way ANOVA was used to compare multiple groups. The significant differences were set as *P*-value < 0.05.

## Results

### duPIAS2 Interacts With DK212 NP

**FIGURE 1:**
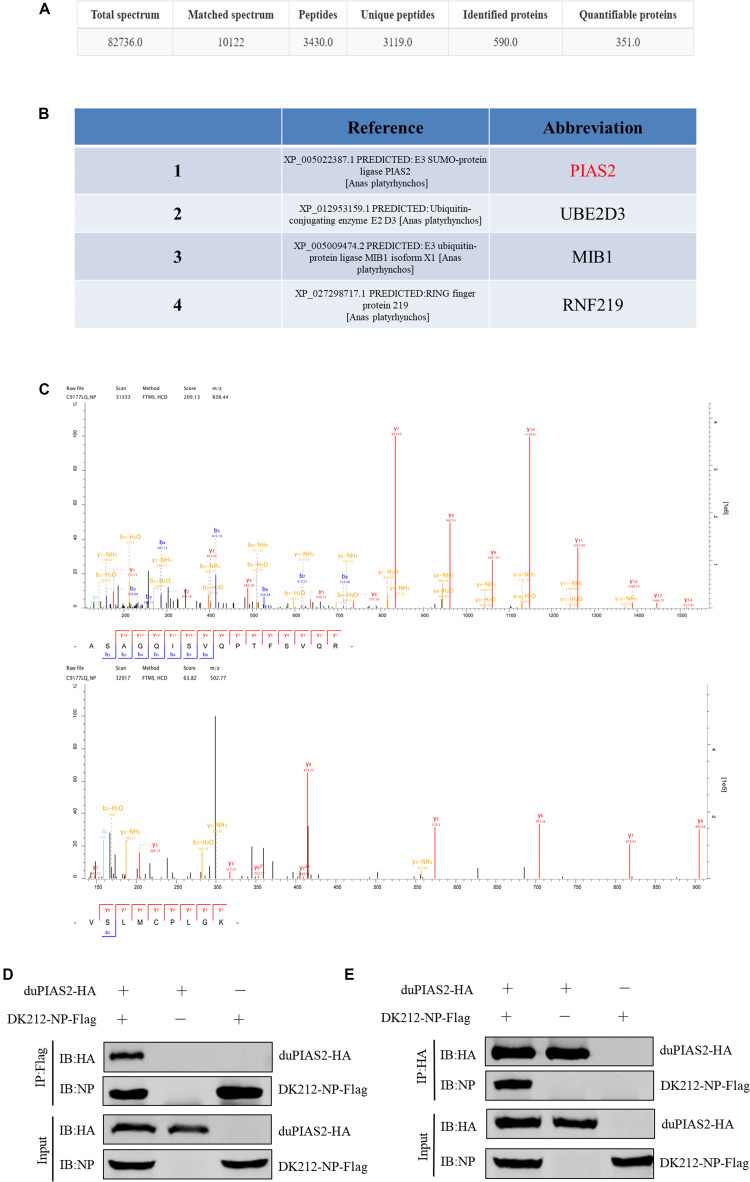
duPIAS2 is identified as a DK212 NP-interacting protein. **(A–C)** pCAGGS-DK212-NP-Flag or the control vector pCAGGS-eGFP-Flag was transfected into DEF cells. After 36 h, the cells were lysed and immunoprecipitated using anti-Flag agarose beads. The immunoprecipitated protein complexes were identified using LC-MS/MS. **(A)** Basic statistics for the LC-MS/MS data from pCAGGS-DK212-NP-Flag group. **(B)** A partial of the identified proteins interacted with DK212 NP using LC-MS/MS. **(C)** Mass spectrometry of the representative peptides of DK212 NP (upper panel) and duPIAS2 (lower panel). **(D,E)** duPIAS2 interacted with DK212 NP in transfected cells. HEK 293T cells were transfected with pCAGGS-duPIAS2-HA and pCAGGS-DK212-NP-Flag. After 36 h, the cells were lysed and immunoprecipitated using anti-Flag agarose beads **(D)** or anti-HA agarose beads **(E)**. The interaction was detected by Western blot using an anti-HA and anti-Flag antibody.

Among influenza A viruses, NPs are conserved structural proteins. To investigate DK212 NP-interacting proteins in DEF cells, we enriched DK212-NP-Flag from transfected DEF cells by immunoprecipitation using anti-Flag agarose beads and applied the LC-MS/MS method to identify immunoprecipitated proteins. As shown in Figure 1A, a total of 590 proteins were identified, in which 351 proteins contained quantitative information. Some proteins in the immunoprecipitated protein complexes associated with PTM, including SUMOyaltion and ubiquitination, were identified and are listed in Figure 1B. Mass spectrometry results of the representative peptides of DK212 NP and SUMO E3 ligase of duPIAS2 are shown in Figure 1C. These results indicate that duPIAS2 was a potential protein interacting with DK212 NP.

To further confirm the interaction between duPIAS2 and DK212 NP, duPIAS2-HA and DK212-NP-Flag were cotransfected into HEK 293T cells to perform an exogenous Co-IP assay. When the lysates were immunoprecipitated with anti-Flag agarose beads, duPIAS2-HA was coimmunoprecipitated with DK212-NP-Flag using an anti-HA antibody (Figure 1D). Meanwhile, when the lysates were immunoprecipitated with anti-HA agarose beads, DK212-NP-Flag was coimmunoprecipitated with duPIAS2-HA using an anti-NP antibody (Figure 1E). Therefore, duPIAS2 was able to interact with DK212 NP in HEK 293T cells by Co-IP assay.

### duPIAS2 Colocalizes With DK212 NP

**FIGURE 2:**
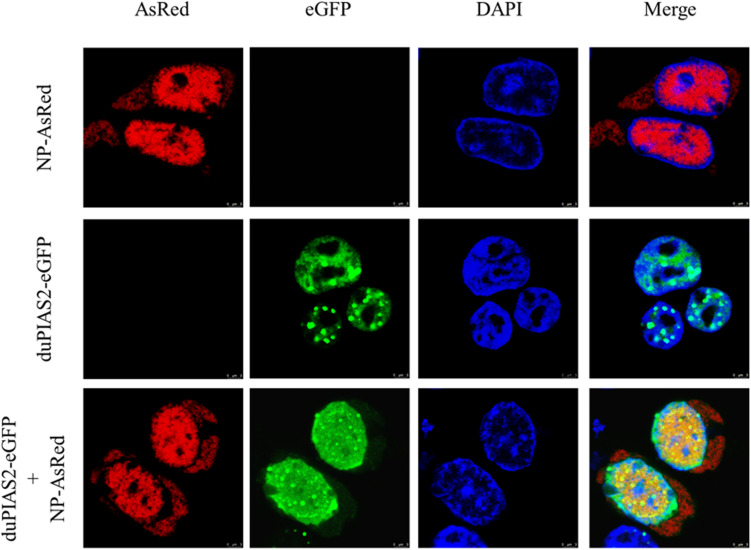
duPIAS2 and DK212 NP were colocalized in transfected cells. HEK 293T cells were cultured on coverslips in 24-well plates until 70% confluence and were transfected with pCAGGS-NP-AsRed and pCAGGS-duPIAS2-eGFP. After 36 h, the transfected cells were fixed with cold methanol, stained with DAPI, and visualized by confocal laser scanning microscope.

To study the colocalization between DK212 NP and duPIAS2, we inserted eGFP gene into pCAGGS-duPIAS2 to construct expressing plasmids of duPIAS2-eGFP with green fluorescence and inserted AsRed gene into pCAGGS-DK212-NP to construct NP-AsRed with red fluorescence, respectively. The colocalization between DK212 NP and duPIAS2 in HEK 293T cells were examined by confocal microscopy. As shown in Figure 2, when duPIAS2-eGFP or NP-AsRed was expressed alone in HEK 293T cells, duPIAS2-eGFP was mainly expressed in the nucleus or aggregated into punctate granula in the nucleus, whereas NP-AsRed showed a diffuse expression throughout the cytoplasm and nucleus. When duPIAS2-eGFP and NP-AsRed were cotransfected, NP-AsRed was observed to mainly colocalize with duPIAS2-eGFP in the nucleus (Figure 2). The results of the confocal microscopy images demonstrated that DK212 NP could colocalize with duPIAS2 in the nucleus.

### The Expression of duPIAS2 in DEF Cells Infected With DK212

**FIGURE 3:**
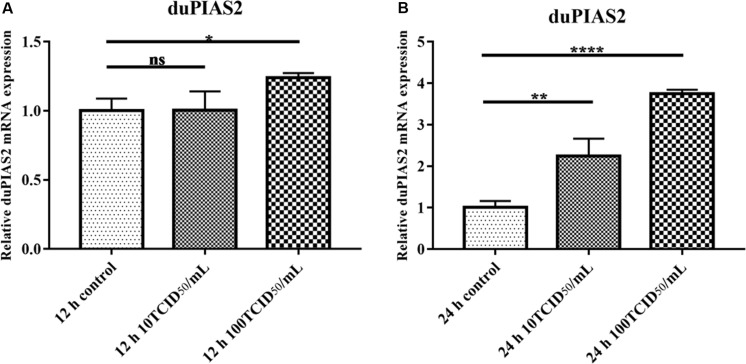
Relative expression of duPIAS2 in DEF cells infected with DK212. DEF cells were cultured in six-well plates until 90% confluence and infected with 10 and 100 TCID_50_/mL of DK212. At 12 h **(A)** and 24 h **(B)** post-infection, the expression of duPIAS2 mRNA was measured by qRT-PCR. duPIAS2 mRNA levels were expressed as relative mRNA indexes, calculated as index (duPIAS2 mRNA copy number/GAPDH mRNA copy number) of test group by index of control group. The experiment was repeated three times. The error bars represent the means and SDs (*n* = 3) and are compared using a student’s *t*-test. ^ns^*P* > 0.05, *P < 0.05; **P < 0.01, ****P < 0.0001.

To investigate the expression of duPIAS2 in DEF cells after infection with H5N1 AIV, DEF cells were infected with DK212 of 10 or 100 TCID_50_/well. When DEF cells were infected with 10 TCID_50_ of DK212, the expression of duPIAS2 hardly changed at 12 hpi but increased twofold at 24 hpi (*P* < 0.01) (Figures 3A,B). When DEF cells were infected with 100 TCID_50_ of DK212, the expression of duPIAS2 was up to 1.2-fold at 12 hpi (*P* < 0.05) and about fourfold at 24 hpi (*P* < 0.0001) (Figures 3A,B). These results show that the expression of PIAS2 in DK212-infected DEF cells significantly increased at 24 hpi, which indicates that DK212 infection could promote the expression of duPIAS2 in DEF cells.

### duPIAS2 Promotes DK212 Replication

**FIGURE 4:**
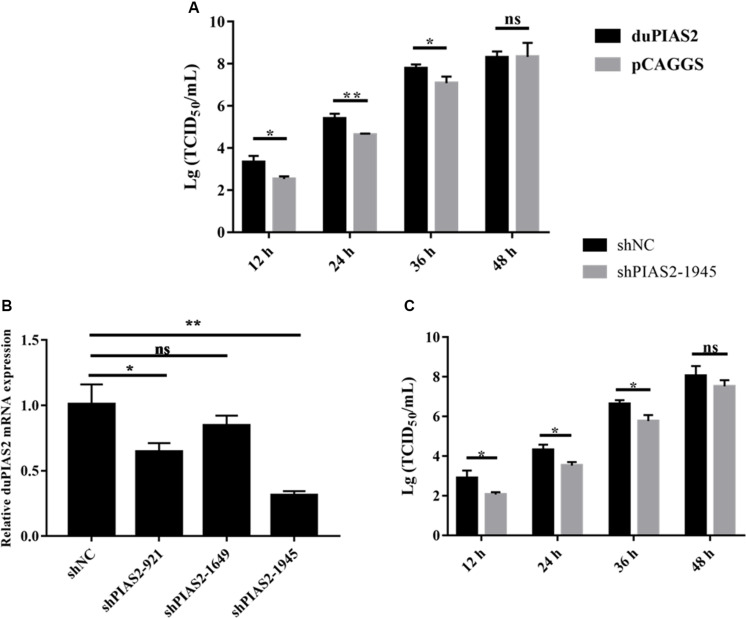
duPIAS2 promotes the replication of DK212. **(A)** DEF cells were transfected with empty vector or pCAGGS-duPIAS2-HA. At 24 h post-transfection, the cells were infected with 100 TCID_50_ of DK212. The culture supernatants were harvested for measurement of the viral TCID_50_ at 12, 24, 36, and 48 h post-infection, respectively. **(B)** DEF cells were transfected with three shRNA targeting duPIAS2 or negative control (shNC). After 36 h, the transfected cells were measured using qRT-PCR for the expression of endogenous duPIAS2. **(C)** DEF cells were transfected with shPIAS2-1945 or shNC. After 24 h, 100 TCID_50_/well DK212 was used to infect shRNA-treated DEF cells. Supernatants were collected at 12, 24, 36, and 48 hpi, and viral titers were measured by TCID50 assay. The experiment was repeated three times. The error bars represent the means and SDs (*n* = 3) and are compared using a student’s *t*-test. ^ns^*P* > 0.05, *P < 0.05; **P < 0.01.

To explore the influence of duPIAS2 overexpression on H5N1 AIV replication, DK212 was used to infect DEF cells, which were transfected with duPIAS2. The titers of DK212 increased from 12 to 48 hpi in cells with or without duPIAS2 overexpression (Figure 4A). DK212 virus titers in duPIAS2-overexpressing cells were higher than that in pCAGGS-transfected cells at 12, 24, and 36 hpi, respectively. The DK212 virus titer in duPIAS2-expressing cells was approximately 6.3 times (*P* < 0.05) higher than that in pCAGGS-transfected cells at 12 hpi and was 5.7 (*P* < 0.01) and 5 times (*P* < 0.05) higher at 24 and 36 hpi, respectively (Figure 4A). These results indicate duPIAS2 could promote DK212 replication.

To further study the effect of duPIAS2 downregulation on DK212 replication, three shRNAs targeting different positions of duPIAS2 were designed and transfected into DEF cells. qRT-PCR assay was used to measure the endogenous expression of duPIAS2 after transfection. As shown in Figure 4B, both shPIAS2-921 and shPIAS2-1945 could inhibit the mRNA expression of PIAS2 in DEF cells. The expression of duPIAS2 in the shPIAS2-1945 group was reduced to 31% (*P* < 0.01) compared with the shNC group. Thus, shPIAS2-1945 was selected for further study. To study the impact of the duPIAS2 knockdown in the replication of DK212, DEF cells were infected with DK212 after being transfected with shPIAS2-1945 or shNC. As shown in Figure 4C, compared with the shNC group, the viral titers in the shPIAS2-1945 group were reduced by 88% (*P* < 0.05), 85% (*P* < 0.05), 85% (*P* < 0.05), and 77% at 12, 24, 36, and 48 h respectively. These results indicate that the knockdown of PIAS2 can inhibit the replication of duPIAS2.

### duPIAS2 Promotes DK212 NP SUMOylation by DK-SUMO1

**FIGURE 5:**
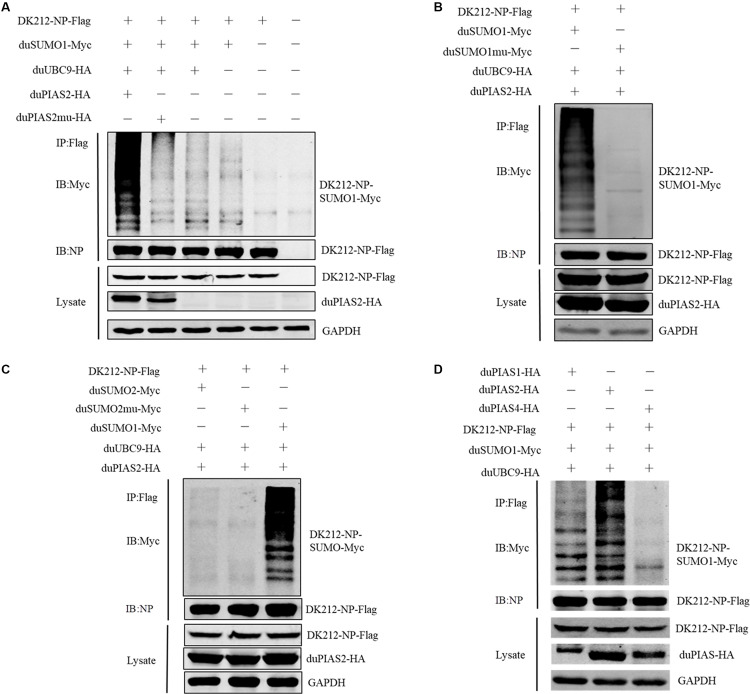
duPIAS2 promotes DK212 NP SUMOylation by duSUMO1. **(A)** HEK 293T cells were transfected with pCAGGS-DK212-NP-Flag, SUMOylation-related expression plasmids, pCAGGS-duPIAS2-HA, or pCAGGS-duPIAS2mu-HA. **(B)** HEK 293T cells were transfected with pCAGGS-DK212-NP-Flag, SUMOylation-related expression plasmids, pCAGGS-duSUMO1-Myc, or pCAGGS-duSUMO1mu-Myc. **(C)** HEK 293T cells were transfected with pCAGGS-DK212-NP-Flag, SUMOylation-related expression plasmids, pCAGGS-duSUMO2-Myc, or pCAGGS-duSUMO2mu-Myc. **(D)** HEK 293T cells were transfected with pCAGGS-DK212-NP-Flag, SUMOylation-related expression plasmids, or pCAGGS-duPIAS-HA. After 36 h, the transfected cells were lysed and immunoprecipitated with anti-Flag agarose beads. The cellular lysates and immunoprecipitation complexes were analyzed by immunoblotting with the indicated antibodies.

To investigate whether the interaction between duPIAS2 and DK212 NP could promote NP SUMOylation, HEK 293T cells were transfected with SUMOylation-related duck expression plasmids together with DK212-NP-Flag or pCAGGS. When duMyc-SUMO1, DK212-NP-Flag, and duUBC9-HA were cotransfected into HEK 293T cells, higher molecular-mass bands were detected by immunoblotting using the anti-Myc antibody, which indicated that NPs were SUMOylated (Figure 5A). Compared with the non-duPIAS2 expression group, the higher-molecular-mass bands were stronger in the duPIAS2 group (Figure 5A). These results indicate that duPIAS2 promoted the SUMOylation of DK212 NP.

There are four SUMO molecules in humans: SUMO1, SUMO2, SUMO3, and SUMO4. GG-to-AA mutations at the C terminus of SUMO could not covalently bind to the target protein (Chen et al., 2004; Gareau and Lima, 2010). To further verify whether duSUMOmu could attach with DK212 NP, duSUMO1mu-Myc, DK212-NP-Flag, duUBC9-HA, and duPIAS2-HA were cotransfected into HEK 293T cells. Interestingly, higher molecular mass bands were not detected in the DK212-NP-Flag immunoprecipitates by immunoblotting (Figure 5B). The results indicated duPIAS2 could not promote duSUMO1mu conjugation to DK212 NP. There are two SUMO isoforms encoded by the duck gene in NCBI, SUMO1, and SUMO2. To verify whether DK212 NP can be modified by duSUMO2, duSUMO2-Myc, duSUMO2mu-Myc, and duSUMO1-Myc were transfected into HEK 293T cells. We found that duSUMO1-Myc readily conjugated to DK212 NP, but duSUMO2-Myc and duSUMO2mu-Myc showed scarcely any conjugation (Figure 5C). To investigate if other duPIAS members could SUMOylate DK212 NP, we transfected duPIAS together with SUMOylation-related duck expression plasmids. Among the PIAS family, we found the higher molecular mass bands were the strongest in the duPIAS2 group and that there were no higher molecular mass bands in the duPIAS4 group (Figure 5D). The results indicated that duPIAS2 promoted DK212 NP SUMOylation specifically by SUMO1.

### K7, K48, and K87 of NP Are Potential Sites to Be SUMOylated

**FIGURE 6:**
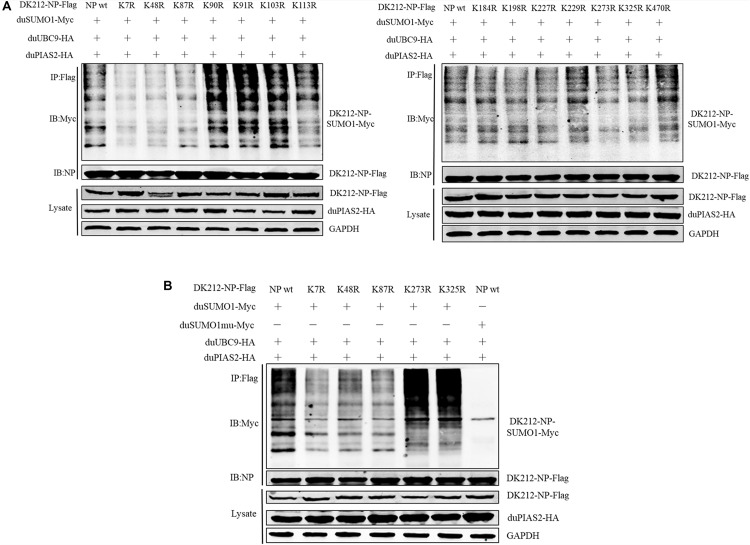
K7, K48, and K87 of DK212 NP were potential SUMOylation site. **(A)** HEK 293T cells were transfected with SUMOylation-related expression plasmids and individual specific K-to-R mutant constructs of NP and NPwt. **(B)** HEK 293T cells were transfected with SUMOylation-related expression plasmids and NP, K7R, K48R, K87R, K273R, or K325R mutant NP to further confirm the SUMOylation sites of NP. After 36 h, the transfected cells were lysed and immunoprecipitated with anti-Flag agarose beads. The cellular lysates and immunoprecipitation complexes were analyzed by immunoblotting with the indicated antibodies.

To further confirm the SUMOylation sites of NP of DK212, all of the 14 lysines in NP were mutated to arginines. These mutated NP, duSUMO1-Myc, duUBC9-HA, and duPIAS2-HA, were cotransfected into HEK 293T cells and then were immunoprecipitated with anti-Flag agarose beads. When the cells were expressed with NP-K7R, NP-K48R, or NP-K87R, the higher-molecular-mass bands were relatively weaker than expressed with wild NP (Figures 6A,B). These results indicated K7, K48, and K87 of NP were potential sites to be SUMOylated.

### The SUMO E3 Activity of duPIAS2 Is Necessary to Promote DK212 Replication

**FIGURE 7:**
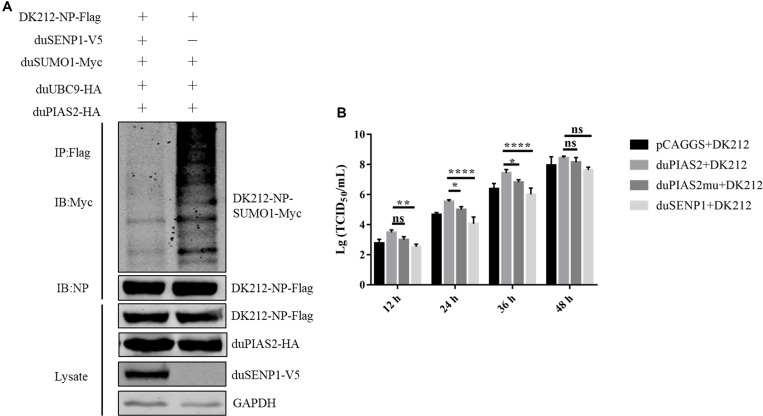
The SUMO E3 activity of duPIAS2 is necessary to promote DK212 replication. **(A)** duSENP1 inhibited the SUMOylation of DK212 NP promoted by duPIAS2. HEK 293T cells were transfected with pCAGGS-DK212-NP-Flag, SUMOylation-related expression plasmids, and pCAGGS-duSENP1-V5. After 36 h, the transfected cells were lysed and immunoprecipitated with anti-Flag agarose beads. **(B)** DEF cells were transfected with pCAGGS-duPIAS2-HA, pCAGGS-duPIAS2mu-HA, pCAGGS-duSENP1-V5, or pCAGGS. At 24 h post-transfection, the cells were infected with 100 TCID_50_ of DK212. The culture supernatants were harvested for measurement of their viral TCID_50_ at 12, 24, 36, and 48 h post-infection, respectively. The experiment was repeated three times. The error bars represent the means and SDs (*n* = 3) and are compared using two-way ANOVA. ^ns^*P* > 0.05, *P < 0.05; **P < 0.01; ****P < 0.0001.

To verify whether the SUMO E3 activity of duPIAS2 was necessary to promote the replication of DK212, HEK 293T cells were transfected with duPIAS2mu-HA (a mutation that lost its SUMO E3 ligase function) or duSENP1-V5 (a deSUMOylation enzyme) together with SUMOylation-related expression plasmids and DK212-NP-Flag. Compared with the duUBC9-Myc + duSUMO1-Myc + DK212-NP-Flag group, the higher molecular mass bands were weaker in the duPIAS2mu-HA group, indicating duPIAS2mu could not promote NP SUMOylation (Figure 5A). Compared with the pCAGGS group, the higher molecular mass bands in the duSENP1-V5 expressed group were weaker, demonstrating that duSENP1 could repress DK212 NP SUMOylation (Figure 7A).

Viral titers were measured by TCID_50_ assay. As shown in Figure 7B, compared with duPIAS2 group, overexpression of duPIAS2mu could not promote the replication of DK212, and the viral titers in the duPIAS2mu group were reduced by 67%, 71% (*P* < 0.05), 75% (*P* < 0.05), and 48% at 12, 24, 36, and 48 h, respectively. Compared with the duPIAS2 group, duSENP1 could not promote the replication of DK212, and the viral titers in the duSENP1 group were reduced by 89% (*P* < 0.01), 97% (*P* < 0.0001), 96% (*P* < 0.0001), and 84% at 12, 24, 36, and 48 h, respectively (Figure 7B). These results indicate that the activity of SUMO E3 of duPIAS2 was important to promote DK212 replication.

## Discussion

In the current study, there were numerous proteins that interacted with DK212 NP in a duck model through LC-MS/MS. And we characterized the functions of duPIAS2, a potential protein interacting with DK212 NP, on influenza virus replication in ducks which are major reservoirs for influenza viruses. We found DK212 infection promoted duPIAS2 expression. PIAS proteins regulate multiple cellular processes, and some PIAS members have a potent capacity to regulate viral replication in mammals. For example, in another study, human PIAS4 was relocated in nuclear domains contained herpes simplex virus (HSV-1) DNA and was found to be synergistic with PML, hence inhibiting HSV-1 replication (Conn et al., 2016). Human PIAS2 was also shown to interact with the Rta of EBV to promote Rta-mediated transcription, which is important in the EBV lytic cycle (Liu et al., 2006). Through PIAS2 overexpression and a knockdown assay, we found that PIAS2 could promote the replication of DK212 in DEF cells. This indicates that PIAS2 is involved in the replication of DK212 in ducks.

It has been reported that human PIAS2α could interact with NP of WSN H1N1 to maintain the normal replication of WSN in mammal cells (Han et al., 2014). Through LC-MS/MS and an exogenous Co-IP assay in transfected cells, we found duPIAS2 could interact with DK212 NP. The PINIT motif of the PIAS family is highly conserved, and it has been implicated in nuclear retention (Shuai and Liu, 2005). NP of the influenza virus contain nuclear localization signals (NLS) that promote importing vRNP into the host nucleus (Portela and Digard, 2002). Our confocal microscopy images indicated duPIAS2 mainly expressed in the nucleus or aggregated into punctate granula in the nucleus. duPIAS2 and DK212 NP could colocalize in the nucleus when coexpressed. These results indicate that duPIAS2 can interact with DK212 NP. We speculate that duPIAS2 might interact with DK212 NP in the nucleus to promote the replication of DK212.

PIAS2 is a characteristic SUMO E3 ligase. It has been reported that human PIAS2 can SUMOylate papillomavirus E1, which is essential for the correct intranuclear localization of E1 (Rosas-Acosta et al., 2005). Here, we found that DK212 NP may be SUMOylated by SUMO1 and is promoted by duPIAS2. The RLD of PIAS2 is involved in the activity of SUMO E3 ligase and is highly conserved across different PIASs (Shuai, 2006). PIAS proteins contain a conserved tryptophan in the middle of their RLD. Mutation of this tryptophan to alanine would eliminate the E3 ligase activity of PIAS (Kotaja et al., 2002). And in human PIAS2α, PIAS2α-W383A could not SUMOylate WSN NP (Han et al., 2014). We found the W374A mutation of duPIAS2 corresponds to W383A of human PIAS2α, and duPIAS2-W374A seems to lose its activity when SUMOylating DK212 NP. These results indicate that duPIAS2 could promote DK212 NP SUMOylation.

Like ubiquitination, the lysines of substrate are target sites for SUMO molecules. The conjugation of SUMO molecule to a target protein occurs in a three-step cascade, and the final step is that the carboxyl group of the glycine residues at the SUMO carboxy terminus forms an isopeptide linkage with the amino group of a lysine residue on the substrate molecule (Geiss-Friedlander and Melchior, 2007). In addition, SUMO-modified proteins contain an acceptor lysine within a ψKX(D/E) consensus motif, where ψ is a large hydrophobic residue, and X is any amino acid followed by an acidic residue (Rodriguez et al., 2001). These residues directly interact with the SUMO E2 and thus play a critical role in regulating the stability of interactions between the E2 enzyme and the substrate (Sampson et al., 2001). In the present study, we found lysines 7, 48, and 87 (K7, K48, and K87) of NP from DK212 are potential sites to be SUMOylated. We speculate that these lysines substitution would disrupt the stability of E2-substrates interaction and affect SUMOylation. It was reported that K254 was a SUMOylation site of the P protein of parainfluenza virus 5 (PIV5) and SUMOylation of the P protein at K254 was important for PIV5 growth. However, P-K254R mutation couldn’t lead to a complete lack of SUMOylation. And the authors speculated there were other SUMOylation sites within the P protein (Sun et al., 2011). We found K7, K48, and K87 of NP were potential sites to be SUMOylated in DK212, and speculated SUMOylation of NP might play essential roles in growth of DK212. The study that a virus expressing non-SUMOylated NP compared with that expressing the wild-type NP for the relationship of SUMOylation of NP with DK212 replication needs further research.

The SUMOylation process could be manipulated by viruses to maintain virus replication during infection. For example, Dengue virus NS5 could be modified by SUMO1 and SUMO2 to maintain its stability and antagonize IFN signaling (Su et al., 2016). To study whether NP SUMOylation promoted by duPIAS2 may be related to its regulatory function in the replication of DK212, we tested the influence of duPIAS2mu on DK212 replication. Compared with duPIAS2, duPIAS2mu could not promote NP SUMOylation and DK212 replication. In mammals, SUMOylation is a reversible process that can be readily reversed by SENP (Yeh et al., 2000). In the current study, we constructed duSENP1 and found duSENP1 could repress NP SUMOylation promoted by duPIAS2. Compared with duPIAS2, duSENP1 could not promote the replication of DK212. Influenza A virus carrying the SUMO-defective matrix protein 1 (M1) produces a lower titer of virus (Wu et al., 2011). In addition, SUMOylation enhances non-structural protein 1 (NS1) stability and thus promotes rapid growth of influenza A virus (Xu et al., 2011). At present, however, the E3 ligase that mediates SUMOylation of NS1 and M1 has not been identified. We found duPIAS2 could promote DK212 NP SUMOylation. There were possibilities that NS1 and M1 would be also SUMOylated by duPIAS2 and that those would be involved into DK212 replication. Therefore, we speculate the promotion effect of duPIAS2 on DK212 replication might be related to the SUMOylation of DK212 NP or other viral proteins enhanced by duPIAS2.

## Conclusion

In conclusion, the function of duPIAS2 on DK212 replication was analyzed in this study. duPIAS2 could promote DK212 replication. duPIAS2 could interact with DK212 NP and promote NP SUMOylation by duSUMO1. Furthermore, the SUMO E3 ligase activity of duPIAS2 was necessary for promoting DK212 replication. However, the mechanisms involved in duPIAS2 to promote DK212 replication need further research.

## Data Availability Statement

All datasets generated for this study are included in the article/Supplementary Material.

## Author Contributions

ML and PJ supervised the whole experiments. SZ and QX performed the practical work and completed the experiments. SZ wrote the whole manuscript. ZH, JZ, WW, WL, ZL, and JH provided help during the experiments. CS helped in improving language expression.

## Conflict of Interest

The authors declare that the research was conducted in the absence of any commercial or financial relationships that could be construed as a potential conflict of interest.

## Abbreviations

ABSL-3: animal biosafety level 3
DEF: duck embryo fibroblast
EBV: Epstein-Barr virus
HCV: Hepatitis C virus
HEK 293T: human embryonic kidney 293T
HPAIV: pathogenic avian influenza virus
LC-MS/MS: liquid chromatography–tandem mass spectrometry
LPAIV: low pathogenic avian influenza virus
MDA5: melanoma differentiation-associated gene 5
NLS: nuclear localization signals
NP: nucleoprotein
PIAS2: protein inhibitor of activated STAT2
PML: promyelocytic leukemia protein
PTM: post-translational modification
RLD: RING-finger-like zinc-binding domain
SPF: specific pathogen free
SUMO: small ubiquitin-related modifier
TCID50: 50% tissue culture infectious dose
vRNP: viral ribonucleoprotein.
