# Supplementary material for: Duck PIAS2 Promotes H5N1 Avian Influenza Virus Replication Through Its SUMO E3 Ligase Activity
Source: Front Microbiol. 2020 Jun 11;11:1246. doi: 10.3389/fmicb.2020.01246 (PMC7300270; doi:10.3389/fmicb.2020.01246)
Supplement: Supplementary file 1 [file Presentation_1.pptx]

## Slide 1
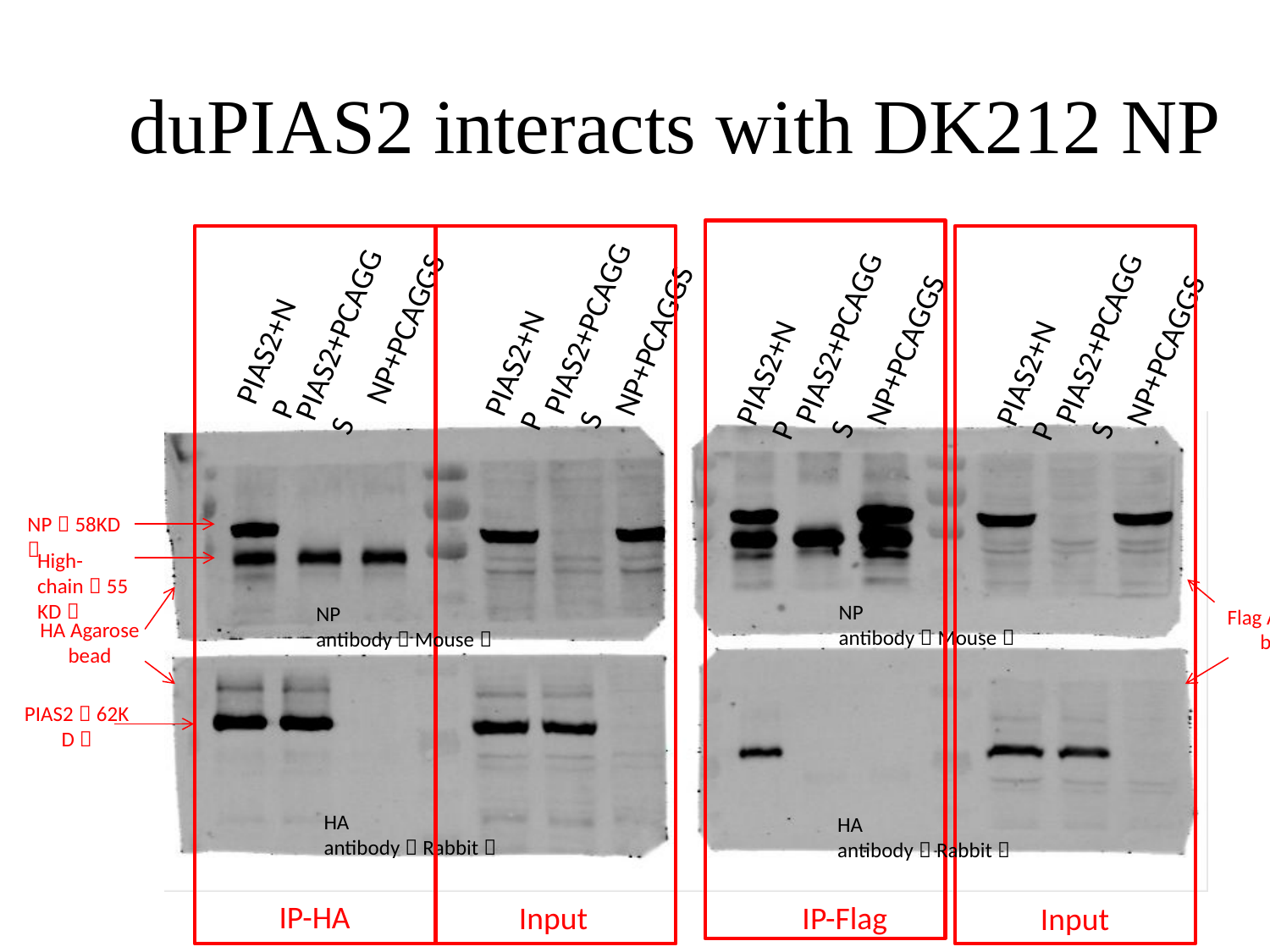

# duPIAS2 interacts with DK212 NP
NP+PCAGGS
PIAS2+PCAGGS
NP+PCAGGS
PIAS2+PCAGGS
PIAS2+PCAGGS
PIAS2+PCAGGS
NP+PCAGGS
NP+PCAGGS
PIAS2+NP
PIAS2+NP
PIAS2+NP
PIAS2+NP
NP（58KD）
High-chain（55KD）
NP antibody（Mouse）
NP antibody（Mouse）
Flag Agarose bead
HA Agarose bead
PIAS2（62KD）
HA antibody（Rabbit）
HA antibody（Rabbit）
IP-HA
Input
IP-Flag
Input

## Slide 2
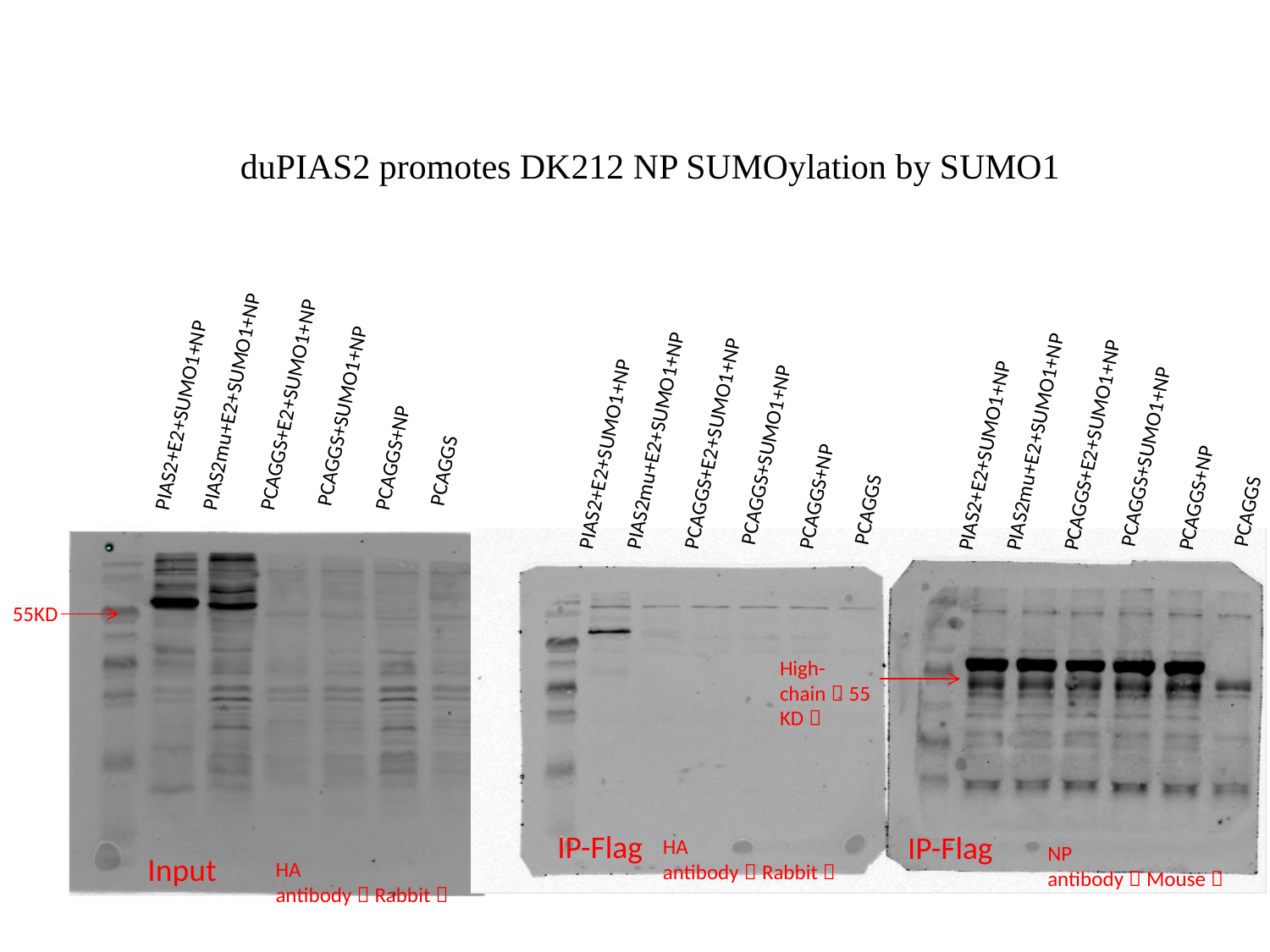

# duPIAS2 promotes DK212 NP SUMOylation by SUMO1
PCAGGS+SUMO1+NP
PCAGGS
PIAS2mu+E2+SUMO1+NP
PCAGGS+E2+SUMO1+NP
PCAGGS+NP
PIAS2+E2+SUMO1+NP
PCAGGS+SUMO1+NP
PCAGGS
PIAS2mu+E2+SUMO1+NP
PCAGGS+E2+SUMO1+NP
PCAGGS+NP
PIAS2+E2+SUMO1+NP
PCAGGS+SUMO1+NP
PCAGGS
PIAS2mu+E2+SUMO1+NP
PCAGGS+E2+SUMO1+NP
PCAGGS+NP
PIAS2+E2+SUMO1+NP
55KD
Input
HA antibody（Rabbit）
High-chain（55KD）
IP-Flag
IP-Flag
HA antibody（Rabbit）
NP antibody（Mouse）

## Slide 3
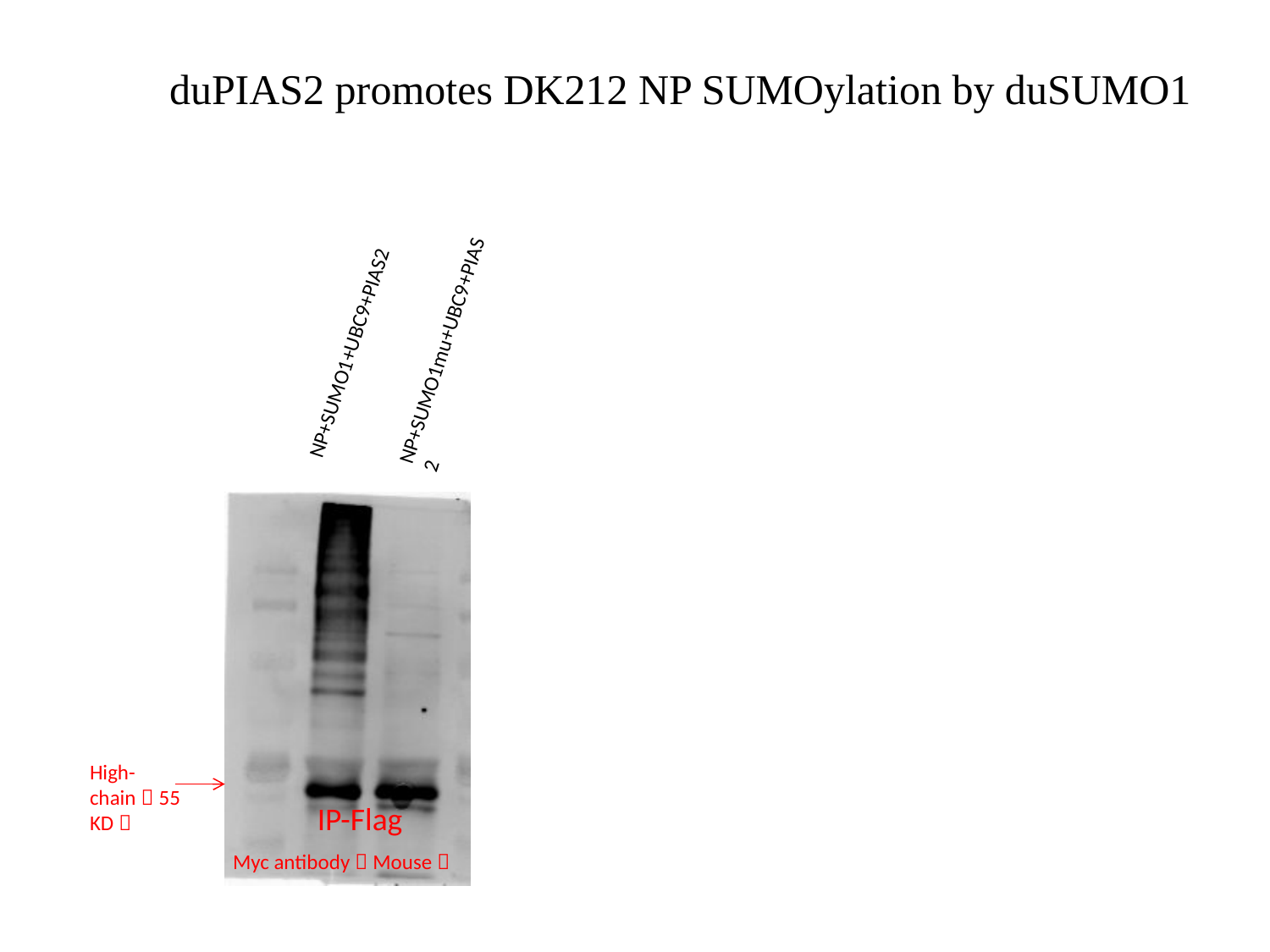

# duPIAS2 promotes DK212 NP SUMOylation by duSUMO1
NP+SUMO1mu+UBC9+PIAS2
NP+SUMO1+UBC9+PIAS2
High-chain（55KD）
IP-Flag
Myc antibody（Mouse）

## Slide 4
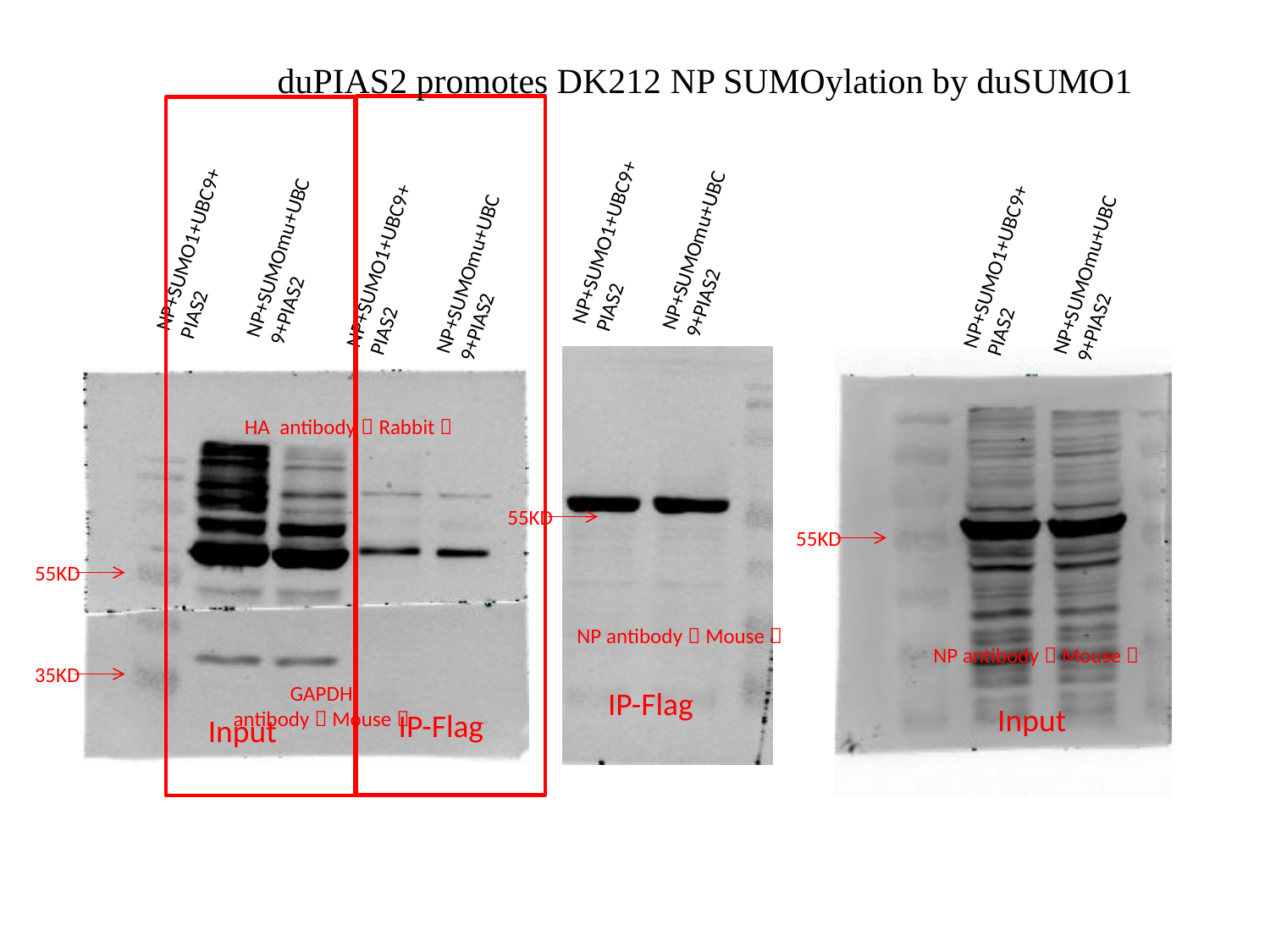

# duPIAS2 promotes DK212 NP SUMOylation by duSUMO1
NP+SUMO1+UBC9+PIAS2
NP+SUMOmu+UBC9+PIAS2
NP+SUMO1+UBC9+PIAS2
NP+SUMOmu+UBC9+PIAS2
NP+SUMO1+UBC9+PIAS2
NP+SUMOmu+UBC9+PIAS2
NP+SUMO1+UBC9+PIAS2
NP+SUMOmu+UBC9+PIAS2
HA antibody（Rabbit）
55KD
55KD
55KD
NP antibody（Mouse）
NP antibody（Mouse）
35KD
GAPDH antibody（Mouse）
IP-Flag
Input
IP-Flag
Input

## Slide 5
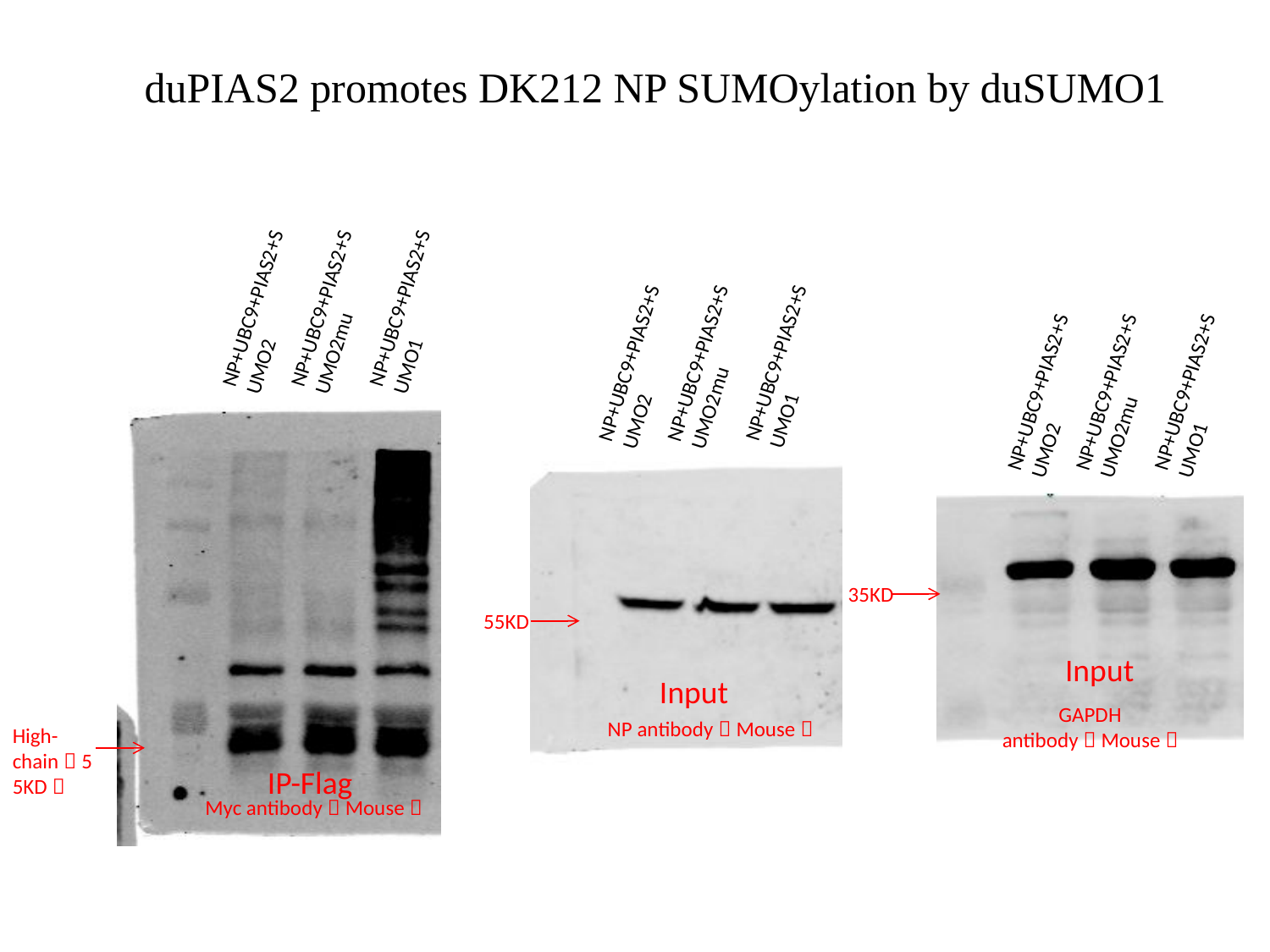

# duPIAS2 promotes DK212 NP SUMOylation by duSUMO1
NP+UBC9+PIAS2+SUMO1
NP+UBC9+PIAS2+SUMO2mu
NP+UBC9+PIAS2+SUMO2
NP+UBC9+PIAS2+SUMO1
NP+UBC9+PIAS2+SUMO2mu
NP+UBC9+PIAS2+SUMO2
NP+UBC9+PIAS2+SUMO1
NP+UBC9+PIAS2+SUMO2mu
NP+UBC9+PIAS2+SUMO2
35KD
55KD
Input
Input
NP antibody（Mouse）
GAPDH antibody（Mouse）
High-chain（55KD）
IP-Flag
Myc antibody（Mouse）

## Slide 6
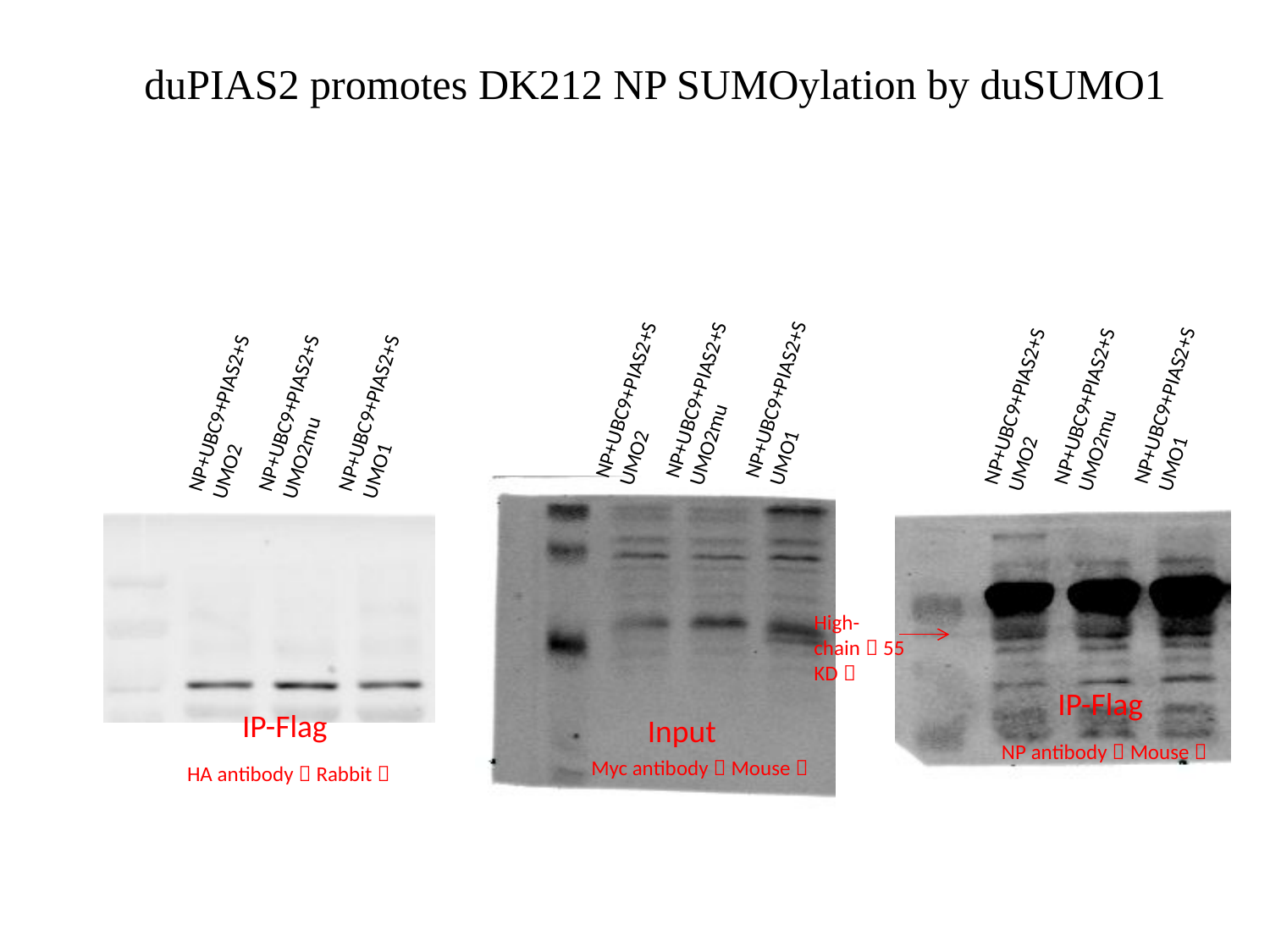

# duPIAS2 promotes DK212 NP SUMOylation by duSUMO1
NP+UBC9+PIAS2+SUMO1
NP+UBC9+PIAS2+SUMO2mu
NP+UBC9+PIAS2+SUMO2
NP+UBC9+PIAS2+SUMO1
NP+UBC9+PIAS2+SUMO2mu
NP+UBC9+PIAS2+SUMO2
NP+UBC9+PIAS2+SUMO1
NP+UBC9+PIAS2+SUMO2mu
NP+UBC9+PIAS2+SUMO2
High-chain（55KD）
IP-Flag
NP antibody（Mouse）
IP-Flag
HA antibody（Rabbit）
Input
Myc antibody（Mouse）

## Slide 7
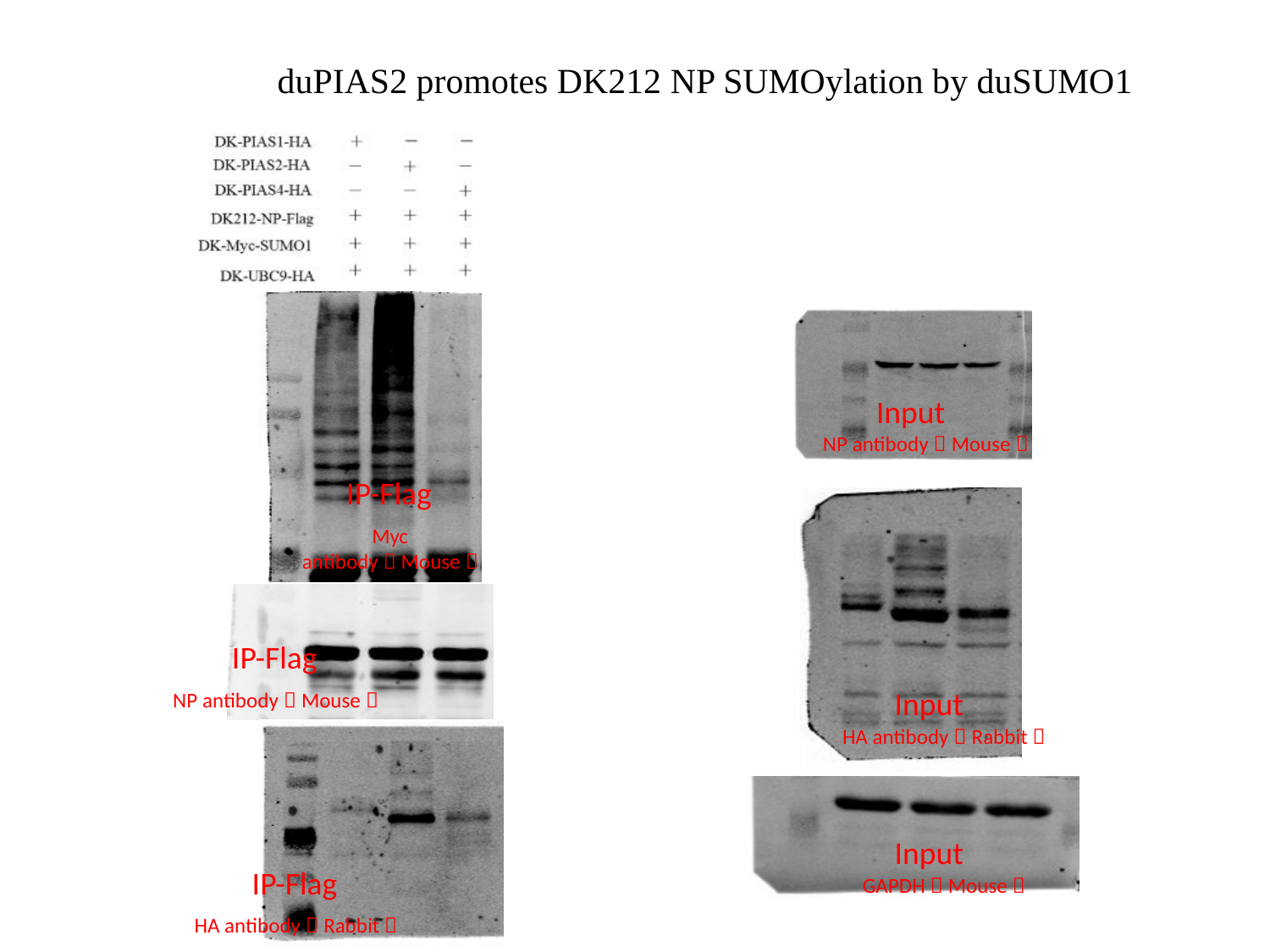

# duPIAS2 promotes DK212 NP SUMOylation by duSUMO1
Input
NP antibody（Mouse）
IP-Flag
Myc antibody（Mouse）
IP-Flag
NP antibody（Mouse）
Input
HA antibody（Rabbit）
Input
GAPDH（Mouse）
IP-Flag
HA antibody（Rabbit）

## Slide 8
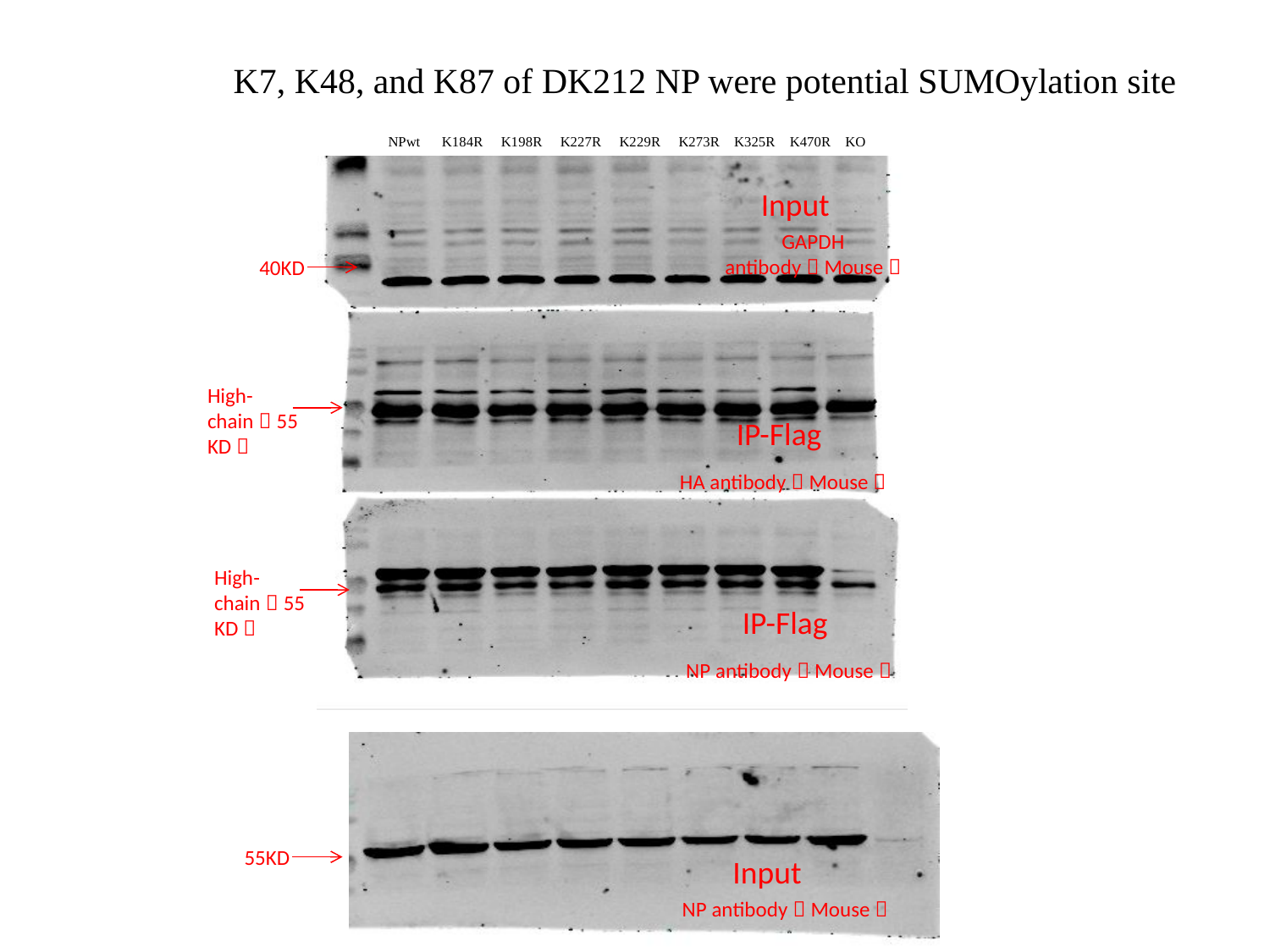

# K7, K48, and K87 of DK212 NP were potential SUMOylation site
 NPwt K184R K198R K227R K229R K273R K325R K470R KO
Input
GAPDH antibody（Mouse）
40KD
High-chain（55KD）
IP-Flag
HA antibody（Mouse）
High-chain（55KD）
IP-Flag
NP antibody（Mouse）
55KD
Input
NP antibody（Mouse）

## Slide 9
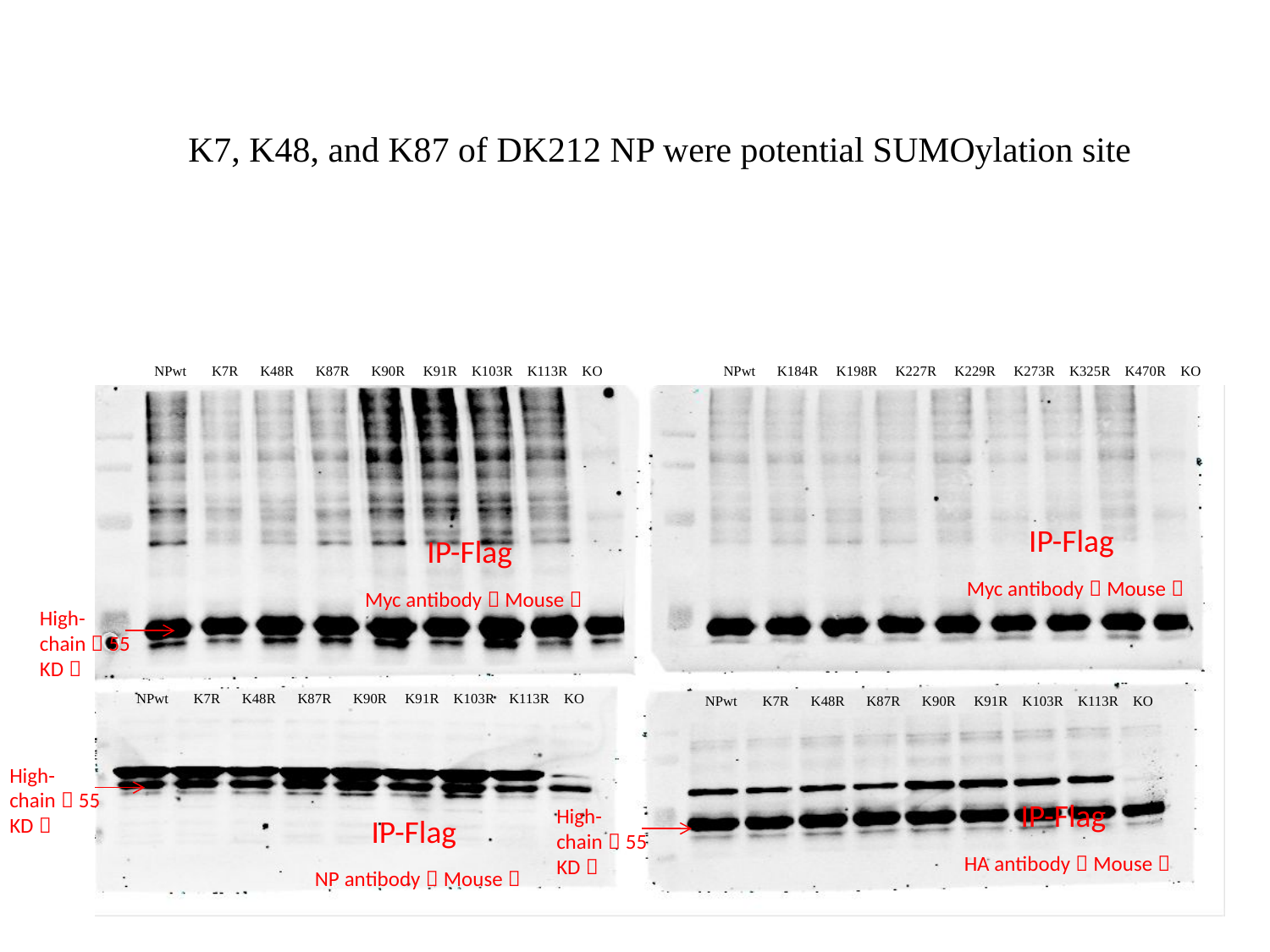

# K7, K48, and K87 of DK212 NP were potential SUMOylation site
 NPwt K7R K48R K87R K90R K91R K103R K113R KO
 NPwt K184R K198R K227R K229R K273R K325R K470R KO
IP-Flag
Myc antibody（Mouse）
IP-Flag
Myc antibody（Mouse）
High-chain（55KD）
 NPwt K7R K48R K87R K90R K91R K103R K113R KO
 NPwt K7R K48R K87R K90R K91R K103R K113R KO
High-chain（55KD）
IP-Flag
HA antibody（Mouse）
High-chain（55KD）
IP-Flag
NP antibody（Mouse）

## Slide 10
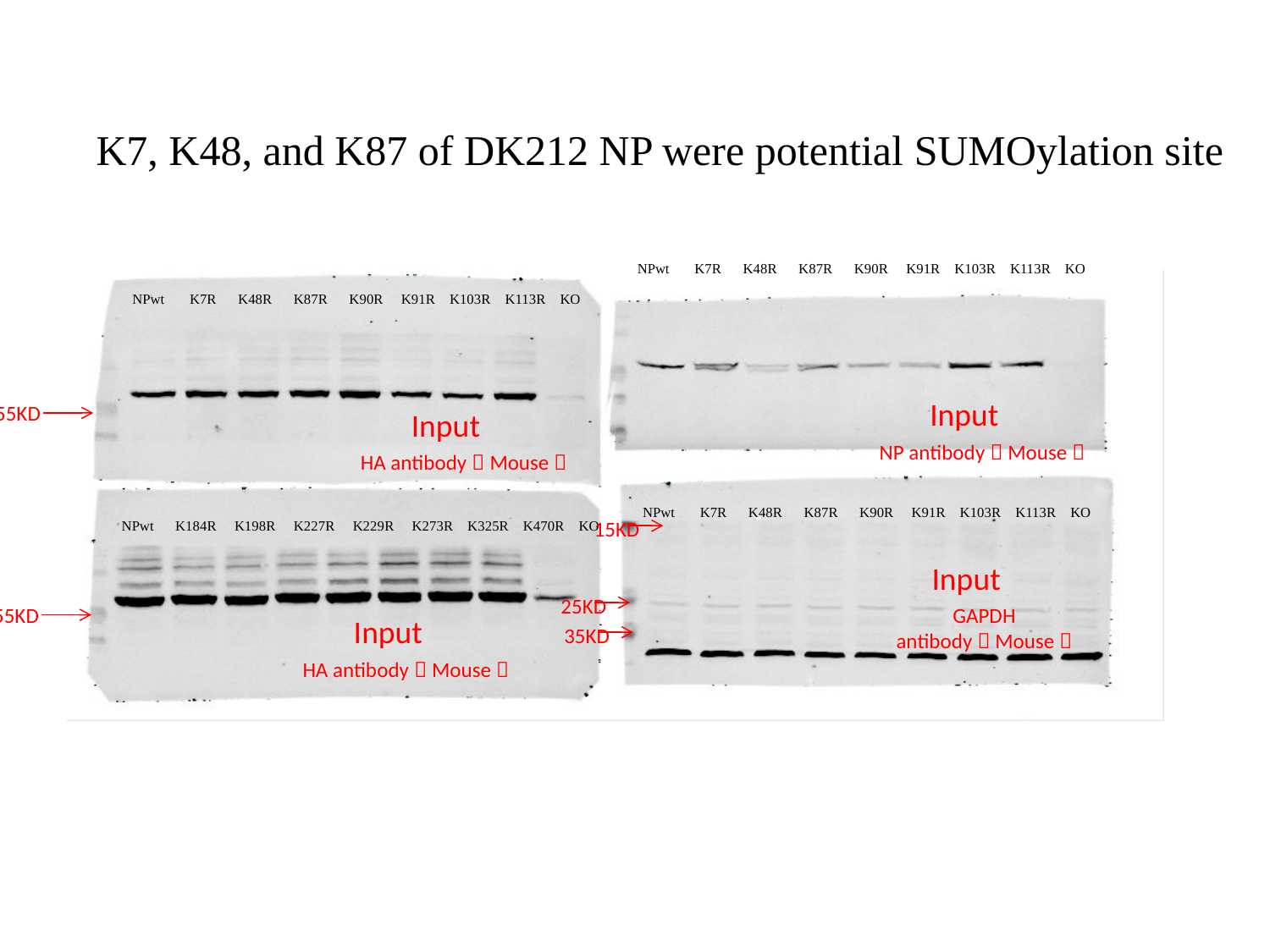

# K7, K48, and K87 of DK212 NP were potential SUMOylation site
 NPwt K7R K48R K87R K90R K91R K103R K113R KO
 NPwt K7R K48R K87R K90R K91R K103R K113R KO
Input
NP antibody（Mouse）
55KD
Input
HA antibody（Mouse）
 NPwt K7R K48R K87R K90R K91R K103R K113R KO
15KD
 NPwt K184R K198R K227R K229R K273R K325R K470R KO
Input
GAPDH antibody（Mouse）
25KD
55KD
Input
HA antibody（Mouse）
35KD

## Slide 11
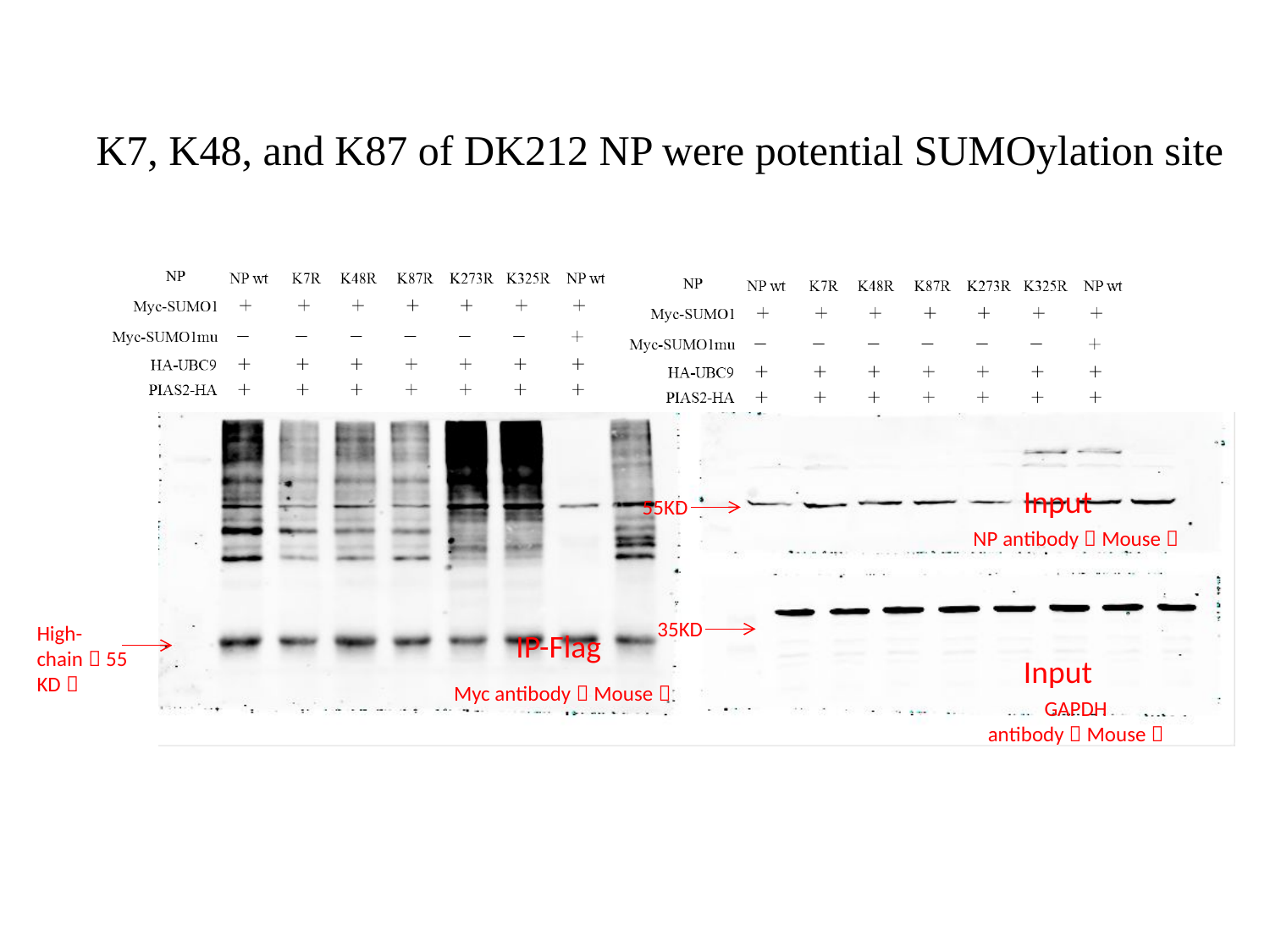

# K7, K48, and K87 of DK212 NP were potential SUMOylation site
Input
NP antibody（Mouse）
55KD
35KD
High-chain（55KD）
IP-Flag
Myc antibody（Mouse）
Input
GAPDH antibody（Mouse）

## Slide 12
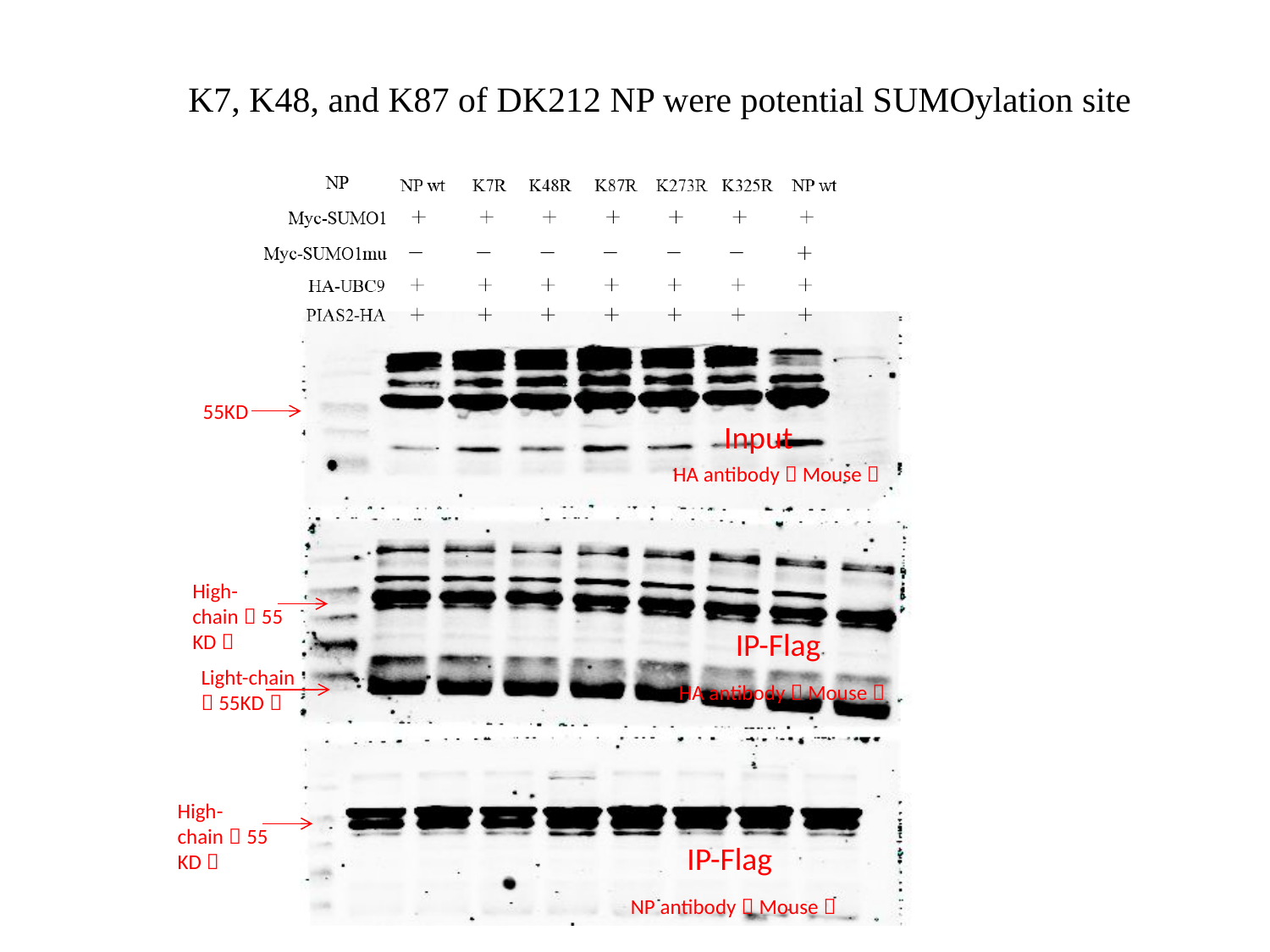

# K7, K48, and K87 of DK212 NP were potential SUMOylation site
55KD
Input
HA antibody（Mouse）
High-chain（55KD）
IP-Flag
HA antibody（Mouse）
Light-chain（55KD）
High-chain（55KD）
IP-Flag
NP antibody（Mouse）

## Slide 13
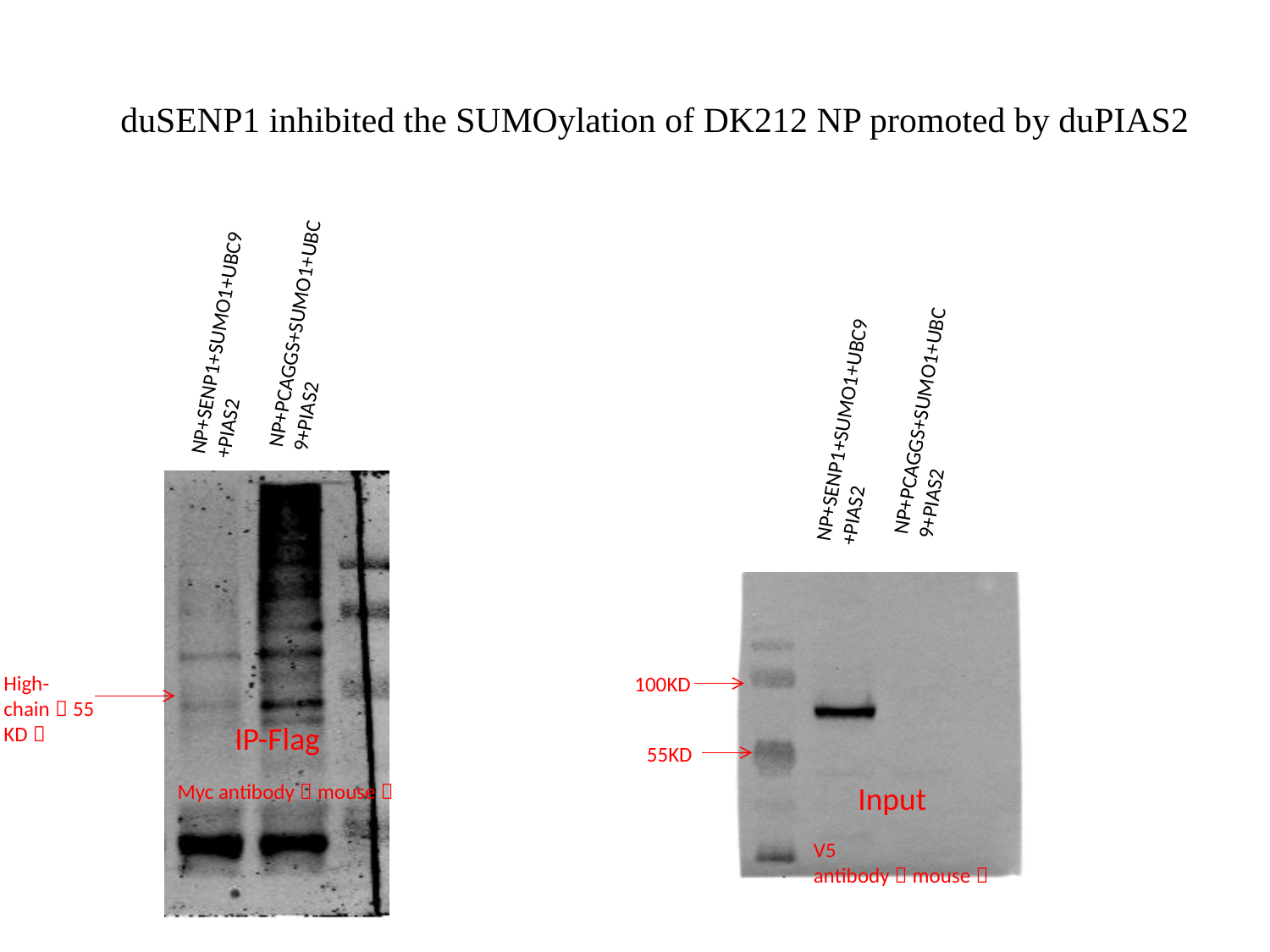

# duSENP1 inhibited the SUMOylation of DK212 NP promoted by duPIAS2
NP+PCAGGS+SUMO1+UBC9+PIAS2
NP+SENP1+SUMO1+UBC9+PIAS2
NP+PCAGGS+SUMO1+UBC9+PIAS2
NP+SENP1+SUMO1+UBC9+PIAS2
High-chain（55KD）
100KD
IP-Flag
55KD
Myc antibody（mouse）
Input
V5 antibody（mouse）

## Slide 14
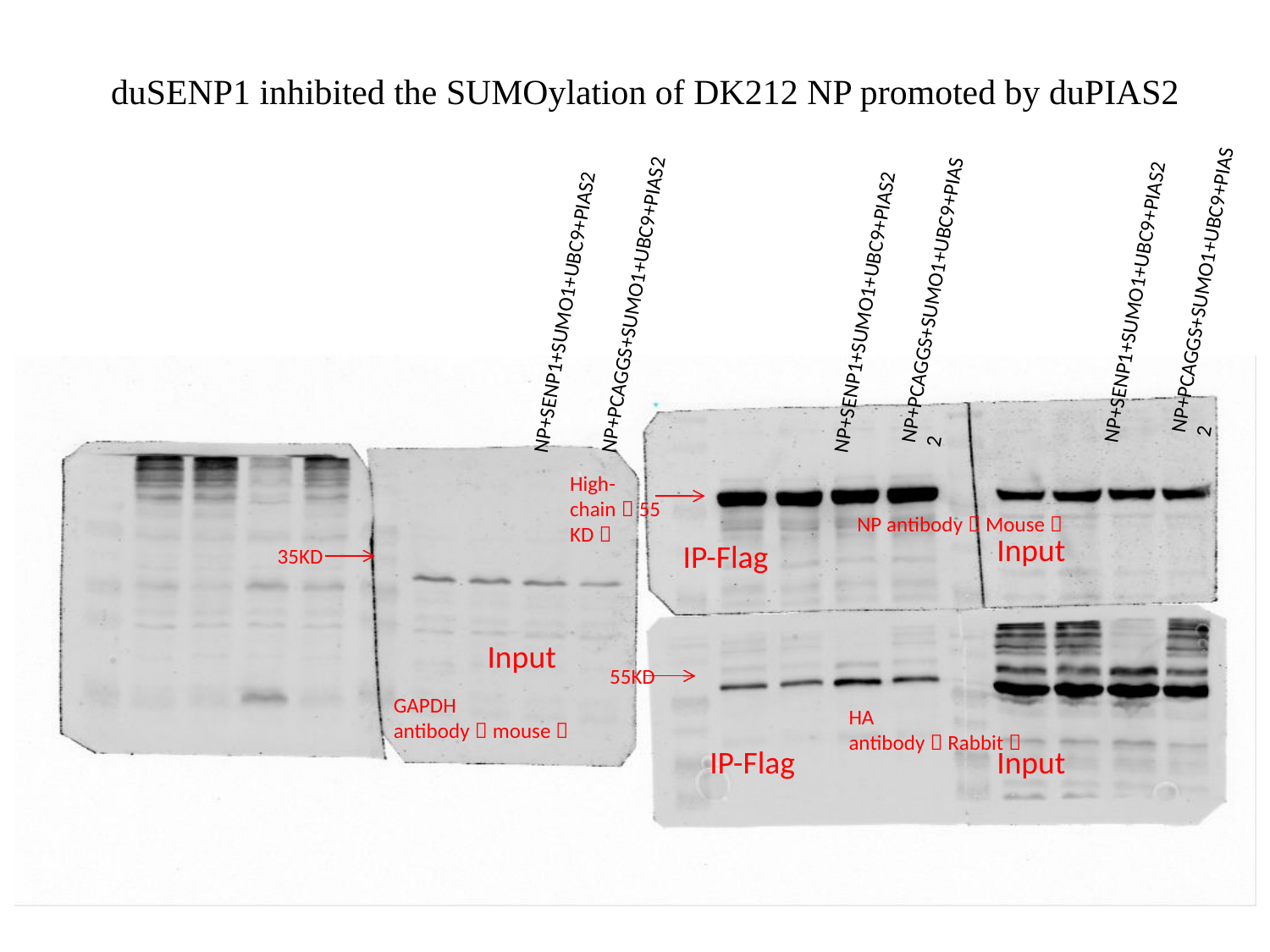

# duSENP1 inhibited the SUMOylation of DK212 NP promoted by duPIAS2
NP+PCAGGS+SUMO1+UBC9+PIAS2
NP+SENP1+SUMO1+UBC9+PIAS2
NP+PCAGGS+SUMO1+UBC9+PIAS2
NP+SENP1+SUMO1+UBC9+PIAS2
NP+PCAGGS+SUMO1+UBC9+PIAS2
NP+SENP1+SUMO1+UBC9+PIAS2
High-chain（55KD）
NP antibody（Mouse）
Input
IP-Flag
35KD
Input
55KD
GAPDH antibody（mouse）
HA antibody（Rabbit）
IP-Flag
Input
